# Diversity of *Colletotrichum* species associated with anthracnose on *Euonymus japonicus* and their sensitivity to fungicides

**DOI:** 10.3389/fpls.2024.1411625

**Published:** 2024-06-13

**Authors:** Yayong Liu, Xiaoqian Tan, Juan Zhao, Yajie Niu, Tom Hsiang, Zhihe Yu, Wentao Qin

**Affiliations:** ^1^ Institute of Plant Protection, Beijing Academy of Agriculture and Forestry Sciences, Beijing, China; ^2^ Beijing Key Laboratory of Environment Friendly Management on Fruit Diseases and Pests in North China, Beijing Academy of Agriculture and Forestry Sciences, Beijing, China; ^3^ College of Life Sciences, Yangtze University, Jingzhou, Hubei, China; ^4^ School of Environmental Sciences, University of Guelph, Guelph, ON, Canada

**Keywords:** *Euonymus japonicus*, *Colletotrichum*, anthracnose, fungicides, pathogenicity

## Abstract

As an evergreen shrub, *Euonymus japonicus* plays a crucial role in urban landscape construction, and its growth is affected by severe foliar anthracnose caused by *Colletotrichum* spp. However, the biodiversity of *Colletotrichum* species associated with anthracnose on *E. japonicus* remains undetermined. This study involved a two-year collection of *E. japonicus* leaf samples with typical anthracnose symptoms from 9 districts in Beijing, China. A total of 194 *Colletotrichum* isolates were obtained, and eight *Colletotrichum* species were subsequently identified using morphological characteristics and molecular identification with the *ACT*, *GADPH*, *CHS*, *TUB2*, and *CAL* genes, as well as the rDNA-ITS region. These species included *Colletotrichum aenigma*, *C. fructicola*, *C. gloeosporioides*, *C. grossum*, *C. hebeiense*, *C. karstii*, *C. siamense*, and *C. theobromicola* with *C. siamense* being the most prevalent (57%), followed by *C. aenigma* and *C. theobromicola*. Furthermore, *C. fructicola*, *C. grossum* and *C. hebeiense* are reported for the first time as causal agents of anthracnose on *E. japonicus* worldwide, and *C. karstii* is newly reported to be associated with *E. japonicus* anthracnose in China. Pathogenicity tests revealed that all tested isolates exhibited pathogenicity in the presence of wounds, emphasizing the need to avoid artificial or mechanical wounds to prevent infection in *E. japonicus* management. The EC_50_ values of five fungicides, namely difenoconazole, flusilazole, tebuconazole, hexaconazole, and prochloraz, were found to be less than 10 mg/L, indicating their strong potential for application. Notably, the EC_50_ of prochloraz was less than 0.05 mg/L for *C. theobromicola*. These findings offer valuable insights for the management of anthracnose on *E. japonicus*.

## 1 Introduction

*Euonymus japonicus* Thunb. plays an important role in urban greening because of its high aesthetic and economic value (Huang et al., 2016). The water retention capacity of *E. japonicus* leaves surpasses that of *Ligustrum vicaryi* among broad-leaved shrub species, thereby enhancing both the aesthetic and functional aspects of landscape through improved soil water storage (Yin et al., 2008). In Beijing, *E. japonicus* is extensively cultivated as a shrub due to its robust resistance to the dry and cold climatic conditions prevalent in the region, effectively reducing particulate matter during winter (Cai et al., 2022). However, *E. japonicus* is highly susceptible to fungal diseases such as powdery mildew and anthracnose (Yao et al., 2018; Lin et al., 2023). The infection of *E. japonicus* caused by *Colletotrichum* spp. leads to the formation of numerous necrotic spots on the leaves, resulting in leaf drying, shedding, and ultimately plant death, significantly impacting its greening effect and landscape aesthetics (Mahoney, 1979; Tan et al., 2023).

*Colletotrichum* spp. have been identified as one of the primary fungal pathogens responsible for anthracnose on a wide range of plants (Dean et al., 2012). The occurrence of anthracnose on *Mangifera indica* in China is caused by *C. fructicola*, *C. gloeosporioides*, *C. karstii*, and *C. siamense*, resulting in damage to leaves and fruits (Li et al., 2019). The occurrence of anthracnose on *Capsicum* spp., caused by *C. viniferum*, results in the formation of sunken, water-soaked necrotic lesions (Diao et al., 2017). However, the identification of *Colletotrichum* is complicated due to the variability in characteristics under different environmental conditions. The traditional identification of *Colletotrichum* species is based on morphological characteristics, including the size and shape of conidia, appressoria, setae, or sclerotia, the presence of sexual structures, as well as cultural attributes such as growth rate and colony color (Hyde et al., 2009). The morphological distinction of *Colletotrichum* species poses a challenge due to their phenotypic similarity and the potential influence of environmental factors on morphological traits. Therefore, a comprehensive integration of available morphological characteristics is commonly employed for systematic discrimination among species (Cai et al., 2009). Currently, a total of 16 *Colletotrichum* species complexes and 15 singleton species have been identified (Sun et al., 2019; Liu et al., 2022). The internal transcribed spacer region (ITS) of ribosomal DNA has been wildly employed for distinguishing *Colletotrichum* species (Freeman et al., 2000). However, relying solely on ITS sequence information is insufficient for resolving species boundaries within the genus (Cannon et al., 2012). Therefore, additional genes such as *ACT*, *CHS-1*, *GAPDH*, and *TUB2* have been employed to elucidate phylogenetic relationships among diverse *Colletotrichum* species (Weir et al., 2012). The integration of molecular sequencing information and morphological data is currently recommended as a comprehensive approach for precise species identification in *Colletotrichum* spp (Cannon et al., 2012; Weir et al., 2012).

In recent years, ten *Colletotrichum* species have been reported to be associated with anthracnose on *E. japonicus*, including *C. aenigma* (Tan et al., 2023), *C. boninense* (Lee et al., 2005), *C. euonymi*, *C. euonymicola* (Lin et al., 2023), *C. gigasporum* (Alizadeh et al., 2015), *C. gloeosporioides* (Huang et al., 2016), *C. karstii* (Alizadeh et al., 2015), *C. siamense* (Wu, 2019), *C. theobromicola* (Qin et al., 2022) and *C. trichellum* (Wu, 1992) in various geographical locations. Anthracnose on *E. japonicus* may be caused by a variety of *Colletotrichum* species, but the specific dominant pathogen and its virulence are still unknown. Recent studies have demonstrated that chemical fungicides (i.e. mancozeb, thiram, ziram, captan, and chlorothalonil) effectively inhibit *Colletotrichum* spp., the causal agents of anthracnose in strawberries (Dowling et al., 2020). However, few data are available about the activity of fungicides in controlling anthracnose of *E. japonicus*, and research is necessary to find the most effective one.

In this study, to elucidate the *Colletotrichum* spp. responsible for anthracnose of *E. japonicus* in a single region, *Colletotrichum* strains were isolated from numerous anthracnose samples of *E. japonicus* in Beijing, China, and identified through morphological analysis, multilocus phylogenetic analysis, and pathogenicity. Through assessing the sensitivity of *Colletotrichum* spp. to multiple fungicides, our objective is to screen and identify efficacious fungicides for anthracnose control in *E. japonicus*. This study will provide a foundation for the development of effective management strategies against this disease.

## 2 Materials and methods

### 2.1 Sampling and fungal isolation

*E. japonicus* leaves with typical anthracnose symptoms were collected from 2020 to 2022 in nine districts of Beijing, China. The tissues were cut into 5 × 5 mm pieces, soaked in 75% ethanol for 10 s and 5% hypochlorite for 1 min, and then washed three times with distilled water for 3 min. The samples were placed on potato dextrose agar (PDA) and incubated at 25°C under a 12 h light/dark cycle. After colonies grew out, the single-spore cultures were obtained and placed on fresh PDA for subsequent analysis. The obtained isolates were deposited in the culture collection of the Institute of Plant Protection, Beijing Academy of Agriculture and Forestry Sciences, China.

### 2.2 DNA extraction, PCR amplification and sequencing

To extract DNA, all the isolates were incubated at 25°C for 6 days on PDA, after which mycelia were collected in 1.5 mL centrifuge tubes. Genomic DNA was extracted using the Solarbio^®^ Fungi Genomic DNA Extraction Kit (Solarbio, China) according to the manufacturer’s instructions. DNA was subsequently detected by electrophoresis with SYBR^®^ Safe DNA gel stain and the qualified DNA were used as templates for PCR amplification. The following primer pairs were used for amplification ITS4/ITS5 (White et al., 1990) for rDNA-ITS; GDF1/GDR1 (Cannon et al., 2012) for glyceraldehyde-3-phospate dehydrogenase (*GADPH*); ACT-512F/ACT-783R (Carbone and Kohn, 1999) for partial actin (*ACT*); T1/Bt2b (Glass and Donaldson, 1995) for β-tubulin (*TUB2*); CHS-79F/CHS-345R (Carbone and Kohn, 1999) for partial chitin synthase (*CHS*); and CL1C/CL2C (Weir et al., 2012) for calmodulin (*CAL*).

The PCR cycles were performed in a C1000 thermal cycler (Bio-Rad) with a total volume of 25 μL. Each PCR system contained 10 μL of 2 × Taq PCR MasterMix, 1 μL of genomic DNA (50 ng/μL), 1 μL each of forward and reverse primers (10 mM) and 7 μL of double-deionized water. The PCR conditions for ITS were as follows: 5 min at 95°C; 35 cycles of 95°C for 30 s, 55°C for 30 s, and 72°C for 45 s; and 10 min at 72°C. The annealing temperatures for the target genes were as follows: *ACT* and *TUB2*, 58°C; *CHS-1*, 56°C; *CAL*, 55°C; and *GAPDH*, 61°C. The remaining PCR conditions for the target genes were the same as those for the ITS region (Li et al., 2023). The genes were sequenced at SinogenoMax Company, Beijing, and aligned with sequences available in the National Center for Biotechnology Information (NCBI) database. Sequences from type or ex-type isolates were selected for further analysis.

### 2.3 Phylogenetic analysis of *Colletotrichum* spp.

The sequences of the *ACT*, *GADPH*, *CHS*, *TUB2*, and *CAL* genes along with the ITS region were combined for the analyses to determine the taxonomic positions of *Colletotrichum* spp. associated with *E. japonicus*. The isolate numbers and the corresponding GenBank accession numbers used for this study were listed in **Supplementary Tables S1** and **S2**. Sequences were assembled and aligned with BioEdit 7.0.5.3 (Hall, 1999). NEXUS files were generated with Clustal X 1.81 (Thompson et al., 1997). Maximum parsimony (MP) analysis was performed using PAUP 4.0b10. The analysis involved running 1000 replicates of a heuristic search, which utilized random sequence addition for initial tree construction followed by tree bisection reconnection branch swapping (Swofford, 2002). All the data were treated as unordered or unweighted, and gaps were treated as missing data. The topological confidence of trees was assessed by the maximum parsimony bootstrap proportion (MPBP) with 1000 replications, each consisting of 10 replicates of randomly adding taxa. The consistency index (CI), homoplasy index (HI), rescaled consistency index (RC), retention index (RI), and tree length (TL) were computed for trees generated using different optimality criteria. The robustness of the most parsimonious trees was assessed by 1,000 bootstrap replications resulting from MP analysis (Hillis and Bull, 1993). The resulting trees were subjected to Kishino-Hasegawa tests to assess their statistical significance (Kishino and Hasegawa, 1989).

Bayesian inference analysis was conducted using MrBayes 3.1.2 (Ronquist and Huelsenbeck, 2003) and a Markov chain Monte Carlo algorithm. The optimal models for nucleotide substitution were determined via statistical selection using MrModeltest v. 2.3 (Nylander, 2004). Simultaneous Markov chains were run for a total of 1,000,000 generations, with each chain initiated from random trees and sampled every 100 generations. After excluding the initial burn-in phase of the analysis consisting of 2,500 trees, the remaining trees were used to compute the Bayesian inference posterior probability. The phylogenetic trees were visualized via TreeviewX v. 0.5.0. The alignments and trees were deposited in TreeBASE.

### 2.4 Morphological identification

Representative isolates of *Colletotrichum* species were selected for morphological identification. Before morphological examination, single-spore isolates were cultured on PDA for 5 days at 25°C. Mycelial discs (6 mm diameter) were cut from colony margins and transferred onto 9-cm-diam Petri dishes, with four plates per isolate. The colony characteristics (color of the upper and lower surfaces) and growth rate (diameters in two perpendicular directions) were recorded after 6 days. The hyphal growth rate (cm/day) was calculated based on the colony diameter. The conidial production, shape and size were examined up to 10 days on PDA incubated at 25°C. The length and width of conidia (60 per isolate) from three-week-old PDA cultures were measured. Conidial appressoria were induced by dropping a conidial suspension (10^6^ conidia/mL; 50 μL) on a concavity slide, which was then placed inside plates containing moistened filter papers, and incubated at 25°C in the dark (Cai et al., 2009). After 48 h, the morphology and dimensions of 50 appressoria were observed microscopically (NIS-Elements F3.0; Nikon) and measured by Image J 1.54d.

### 2.5 The prevalence of *Colletotrichum* species

After morphological and molecular identification, the prevalence of *Colletotrichum* species sampled in different districts in Beijing, China was calculated. The isolation rate (IR) was calculated for each species with the formula, IR % = (NS/NI) × 100, where NS is the number of isolates from the same species, and NI is the total number of isolates from each district (Vieira et al., 2014; Wang et al., 2016). The overall IR was calculated using the NI value equal to the total number of isolates obtained from *E. japonicus* leaves.

### 2.6 Pathogenicity tests of *Colletotrichum* spp.

Representative isolates displaying typical characteristics of *Colletotrichum* species were selected for pathogenicity tests by inoculating mycelial discs and conidial suspensions on healthy, detached leaves of *E. japonicus*. Each treatment included three replicates, with three leaves per replicate. The healthy apical leaves of *E. japonicus* were collected and washed three times with sterile water at the tip of the blade, followed by air-drying on sterilized filter paper. After sterilization, the leaves were punctured three times with a sterile insect pin (0.5 mm in diameter) for inoculation or left untreated (unwounded). To assess pathogenicity, a 6 mm diameter disc with either 20 μL of a 6-day-old culture or a conidial suspension at a concentration of 10^6^ conidia/mL was inoculated onto the left side of each leaf. An equivalent volume of sterile water was used as a parallel control. Individual leaves were placed in plastic cases with distilled water to maintain > 90% relative humidity. They were then incubated at 25°C under a 12/12 h light/dark photoperiod. Daily observations were made to track symptom development. To assess the pathogenicity of various *Colletotrichum* spp., we measured the mean lesion length on *E. japonicus* leaves at 7 days post-inoculation. Infection rates were determined using the following formula: infection rate = (number of infected leaves/number of inoculated leaves) (Fu et al., 2019). The fungus was re-isolated from lesions and recognized by morphological characteristics and multi-gene phylogenetic analysis to fulfill Koch’s postulates.

### 2.7 Sensitivity assessments of the dominant *Colletotrichum* species to fungicides

Mycelial discs of the dominant *Colletotrichum* species (*C. siamense*, *C. aenigma*, and *C. theobromicola*) were inoculated onto PDA, both with and without fungicide amendments. Mycelial discs (6 mm diameter, from PDA culture grown at 25°C and incubated for up to 5 days) were placed onto PDA with or without (control) fungicide amendments. The final concentrations of eight fungicides (difenoconazole, flusilazole, tebuconazole, hexaconazole triadimefon, prochloraz, captan, and chlorothalonil) in the media are shown in **Supplementary Table S3**. Each treatment was tested four times, and the entire experiment was repeated twice. The mean colony diameter was measured at 25°C and incubated up to 5 days under a 12/12 h light/dark photoperiod. The formula for percent inhibition was [(radial growth of the control – radial growth at fungicide concentration)/radial growth of the control- diameter of the control] × 100% (Yan et al., 2014). The log10 probability conversion of the percentage of inhibition of fungicide concentrations was regressed to estimate half of the maximal effective concentration (EC_50_).

### 2.8 Statistical analysis

Data are expressed as mean ± standard deviation unless otherwise mentioned. Data were analyzed and imaged using GraphPad Prism 8.0 and OriginPro 2021 software. Differences between groups were analyzed with one-way ANOVA. The significant difference was defined at P < 0.05.

## 3 Results

### 3.1 Field survey, fungal isolation and prevalence of *Colletotrichum* species

**Figure 1:**
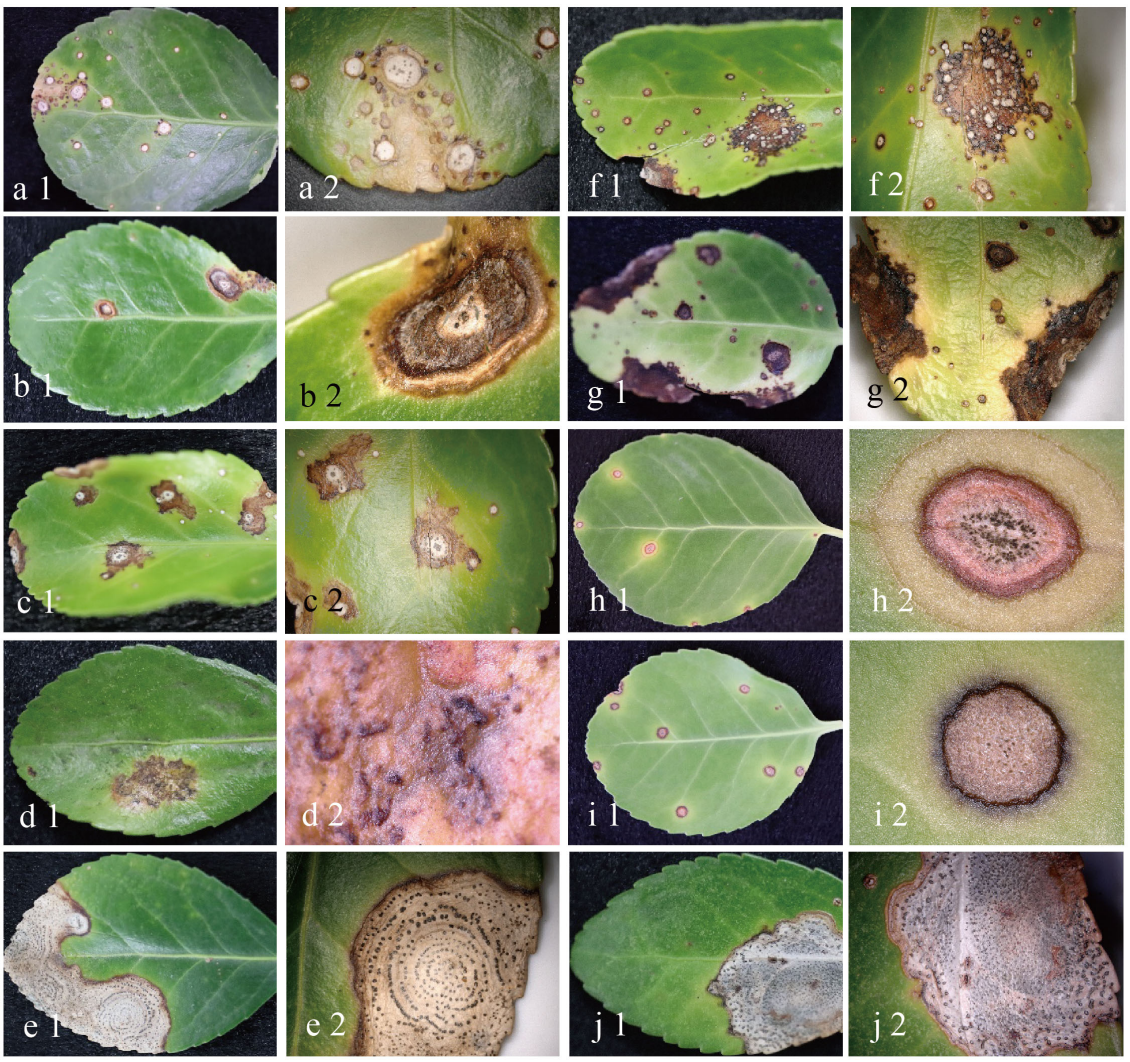
Typical symptoms of anthracnose on leaves of *E. japonicus* at early and late stage in the field. **(A1–2)** symptom I; **(B1–2)** symptom II; **(C1–2)** symptom III; **(D1–2)** symptoms IV; **(E1–2)** symptom V; **(F1–2)** symptom VI; **(G1–2)** symptom VII; **(H1–2)** symptoms VIII; **(I1–2)** symptom VIIII; **(J1–2)** symptom X.

A total of 308 isolates were obtained from 274 leaf samples collected from 9 districts spanning over 55 locations in Beijing, China, between 2020 and 2022. Through the integrated recognition of morphological characteristics and basic sequences blast and analysis, 194 *Colletotrichum* isolates were obtained. Combined with leaves and isolates characteristic identification, symptoms of anthracnose on *E. japonicus* leaves were classified into ten main types (**Figure 1**). Notably, some symptoms in the field may be attributable to the combination of two or three different *Colletotrichum* species (**Supplementary Table S4**), indicating the diversity of anthracnose pathogens of *E. japonicus* and the interactions of multiple *Colletotrichum* species in co-infections.

**Figure 2:**
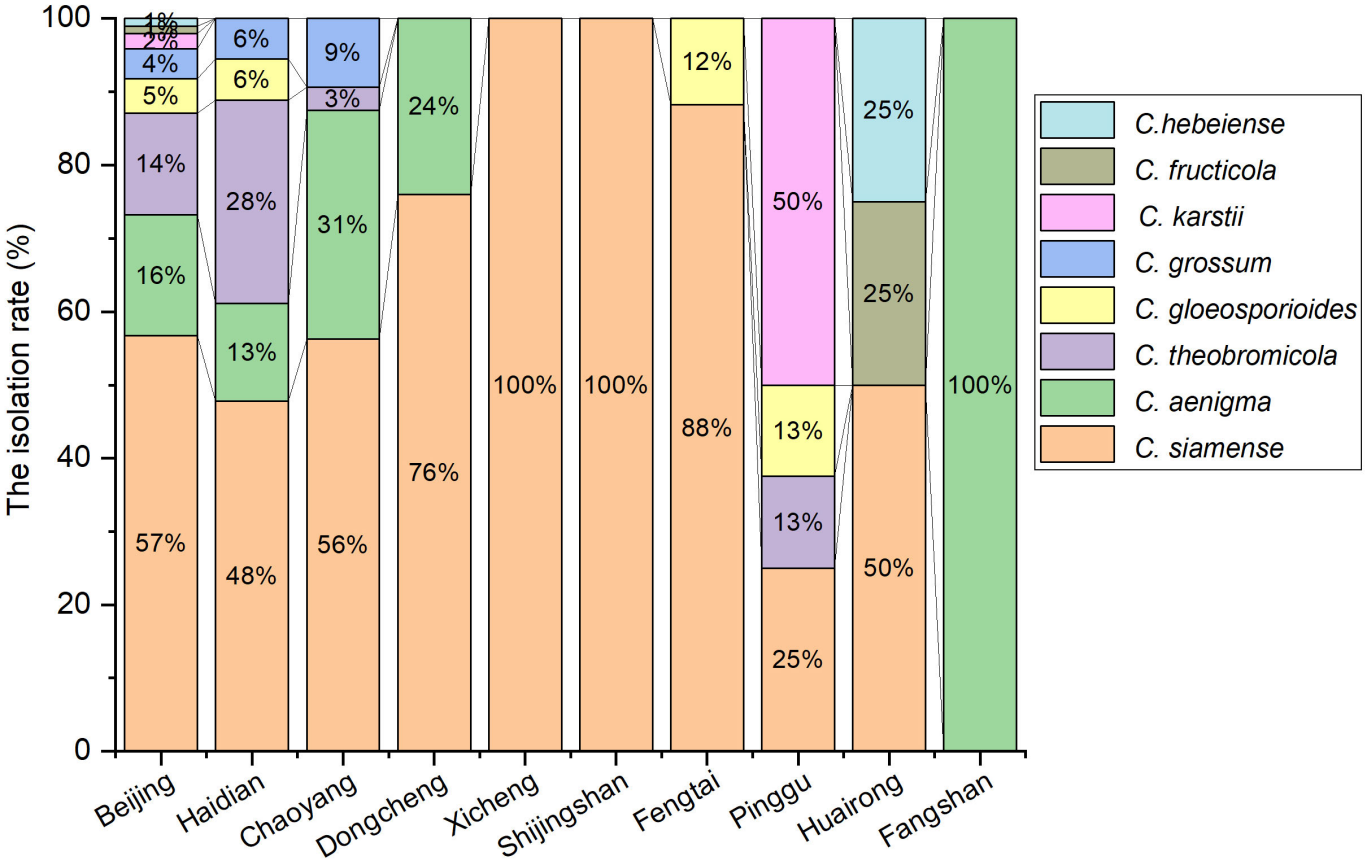
Isolation rate (%) of *Colletotrichum* spp. from the anthracnose leaves of *E. japonicus* in different districts of Beijing, China.

The 194 isolates were identified as eight *Colletotrichum* species (**Figure 2**), including 110 isolates of *C. siamense* (56.7%), 32 isolates of *C. aenigma* (16.5%), 27 isolates of *C. theobromicola* (13.9%), 9 isolates of *C. gloeosporioides* (4.6%), 8 isolates of *C. grossum* (4.1%), 4 isolates of *C. karstii* (2.1%), 2 isolates *C. fructicola* (1.0%) and 2 isolates of *C. hebeiense* (1.0%). Analysis of the diversity and prevalence of *Colletotrichum* species revealed that *C. siamense*, with a prevalence of 56.7%, was the most widespread *Colletotrichum* spp. across eight districts, including Haidian, Chaoyang, Dongcheng, Xicheng, Fengtai, Pinggu, Huairou and Shijingshan districts (**Figure 2**). *C. aenigma* and *C. theobromicola* were less dominant than *C. siamense*, whereas *C. gloeosporioides*, *C. grossum*, *C. karstii*, *C. fructicola*, and *C. hebeiense* were found to be least prevalent. The diversity of *Colletotrichum* species associated with anthracnose of *E. japonicus* exhibited local variations within Beijing. For example, *C. fructicola* and *C. hebeiense* were exclusively isolated from diseased leaves of *E. japonicus* in Huairou district, while *C. karsti* was solely found from Pinggu district. Additionally, *C. grossum* was exclusively identified from Haidian and Chaoyang districts.

### 3.2 Phylogenetic analysis

**Figure 3:**
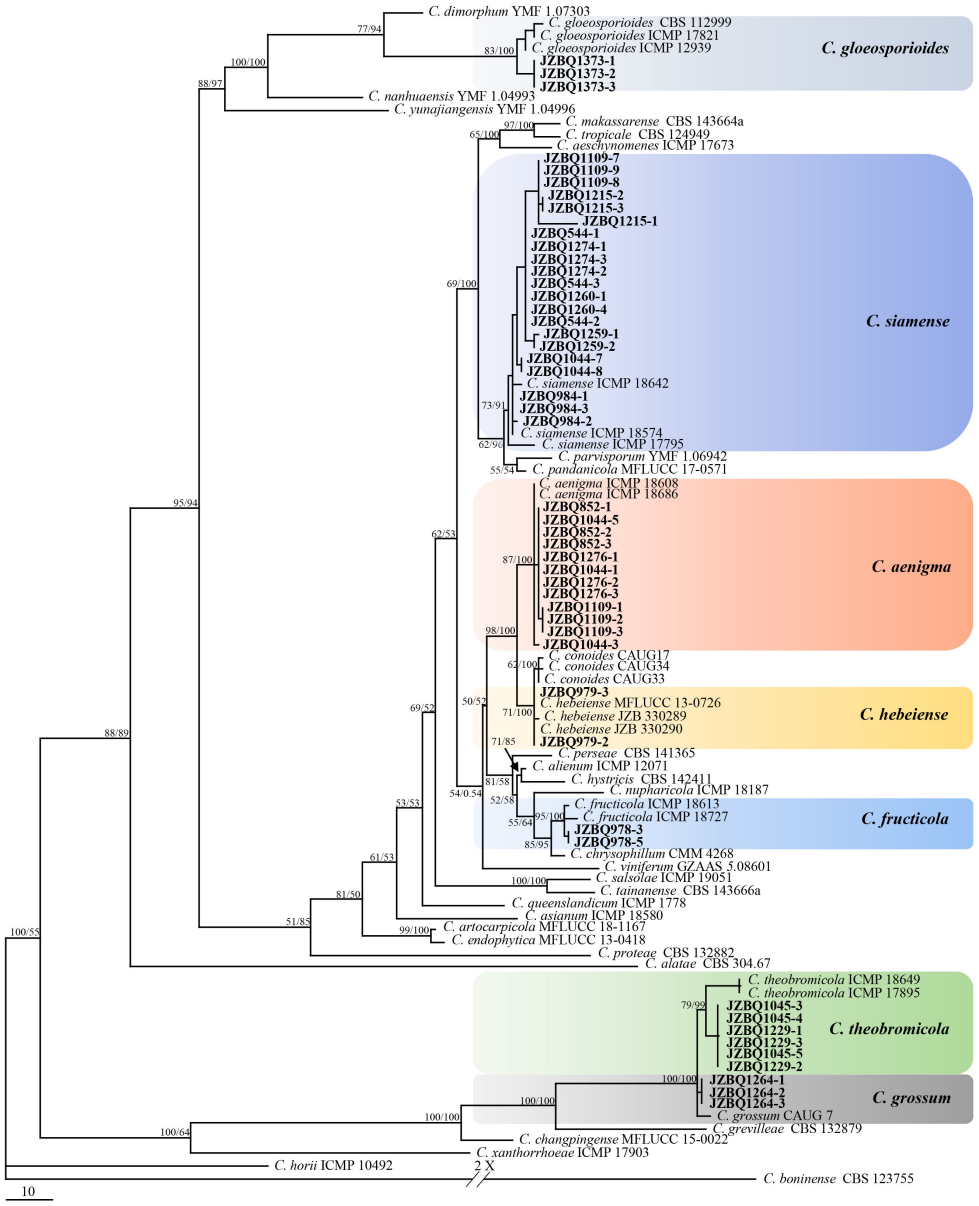
Maximum-parsimony (MP) phylogram reconstructed from the combined sequences of *ITS*, *GAPDH*, *CAL*, *ACT*, *CHS-1* and *TUB2*. *Colletotrichum boninense* (CBS 123755) was selected as the outgroup. MP bootstrap support values (MP ≥ 50%) and bayesian posterior probability (PP ≥ 90%) were shown above the nodes (MP/PP). The scale bar indicates 10 expected changes per site. Isolates in this study were indicated in bold.

**Figure 4:**
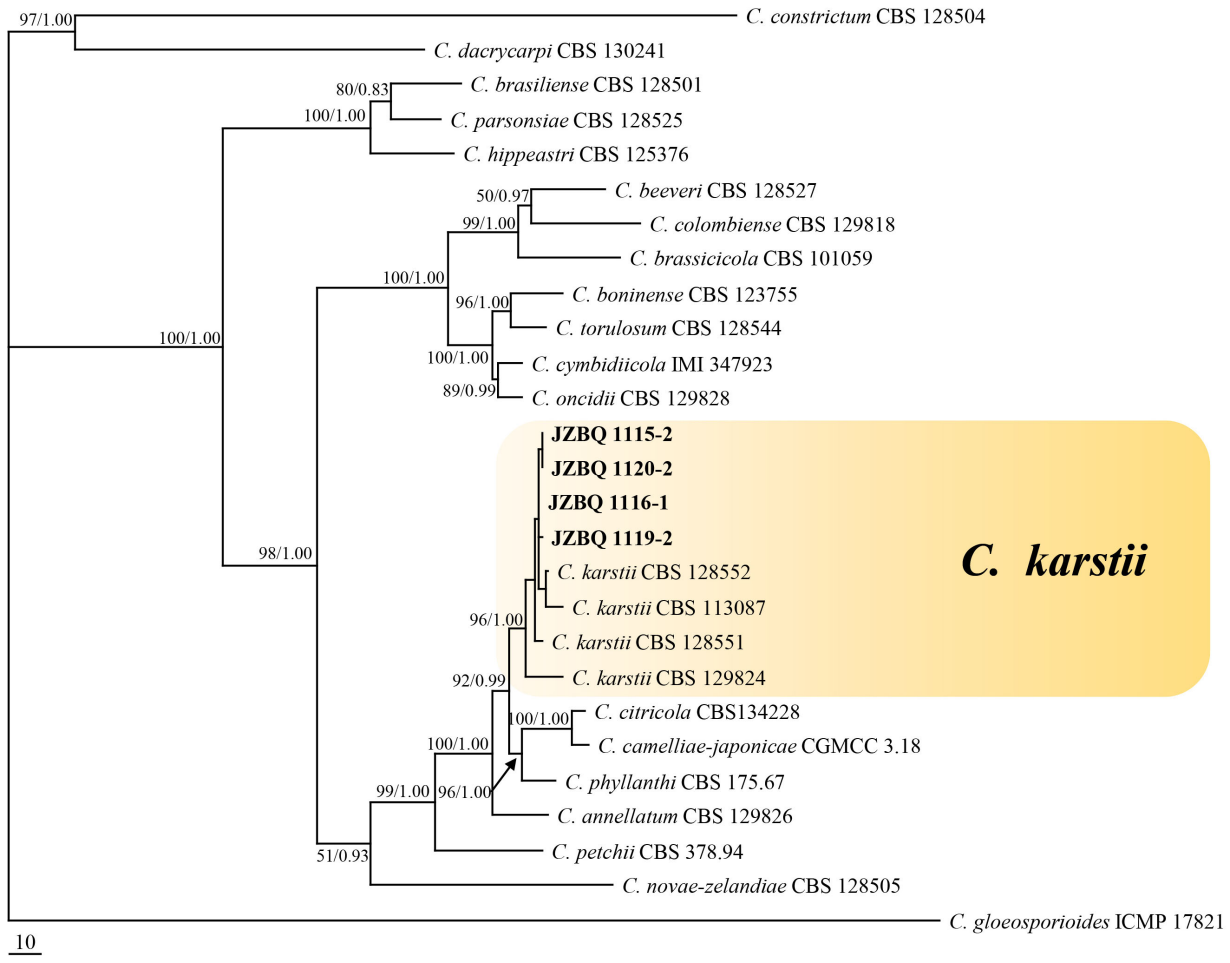
Maximum- parsimony (MP) phylogram reconstructed from the combined sequences of *ITS*, *GAPDH*, *CAL*, *ACT*, *CHS-1* and *TUB2*. *Colletotrichum gloeosporioides* (IMI 356878) was selected as the outgroup. MP bootstrap support values (MP ≥ 50%) and bayesian posterior probability (PP ≥ 90%) were shown above the nodes (MP/PP). The scale bar indicates 10 expected changes per site. Isolates in this study were indicated in bold.

Representative 53 isolates were chosen for phylogenetic analysis based on various sources and *Colletotrichum* species to determine the taxonomic status of *Colletotrichum* isolates. The isolates, along with 66 reference isolates, were selected for analysis based on their combined sequences of the *ACT*, *GADPH*, *CHS*, *TUB2*, and *CAL* genes and rDNA-ITS using maximum parsimony and Bayesian inference methods (**Supplementary Table S1**, **S2**). The isolates could be classified into two *Colletotrichum* species complexes, *C. gloeosporioides* species complex (49 isolates) and *C. boninense* species complex (4 isolates). The *C. gloeosporioides* species complex contained seven species, including *C. aenigma*, *C. fructicola*, *C. gloeosporioides*, *C. grossum*, *C. hebeiense*, *C. siamense* and *C. theobromicola*. A phylogenetic tree was constructed for *C. gloeosporiodes* complex species using *C. boninense* CBS 123755 as an outgroup (tree length = 1217, CI = 0.7527, HI = 0.2473, RC = 0.6859 and RI = 0.9113), which formed four major clades (**Figure 3**). The phylogenetic tree of the *C. boninense* species complex was constructed using *C. gloeosporioides* ICMP 17821 as an outgroup (tree length = 1182, CI = 0.7783, HI = 0.2217, RC = 0.6211, and RI = 0.7980), which also formed four clades (**Figure 4**). The above findings suggest that the majority of pathogenic fungi causing *E. japonicus* anthracnose belong to the *C. gloeosporioides* species complex and exhibit a close evolutionary relationship.

### 3.3 Morphological and culture characteristics

**Figure 5:**
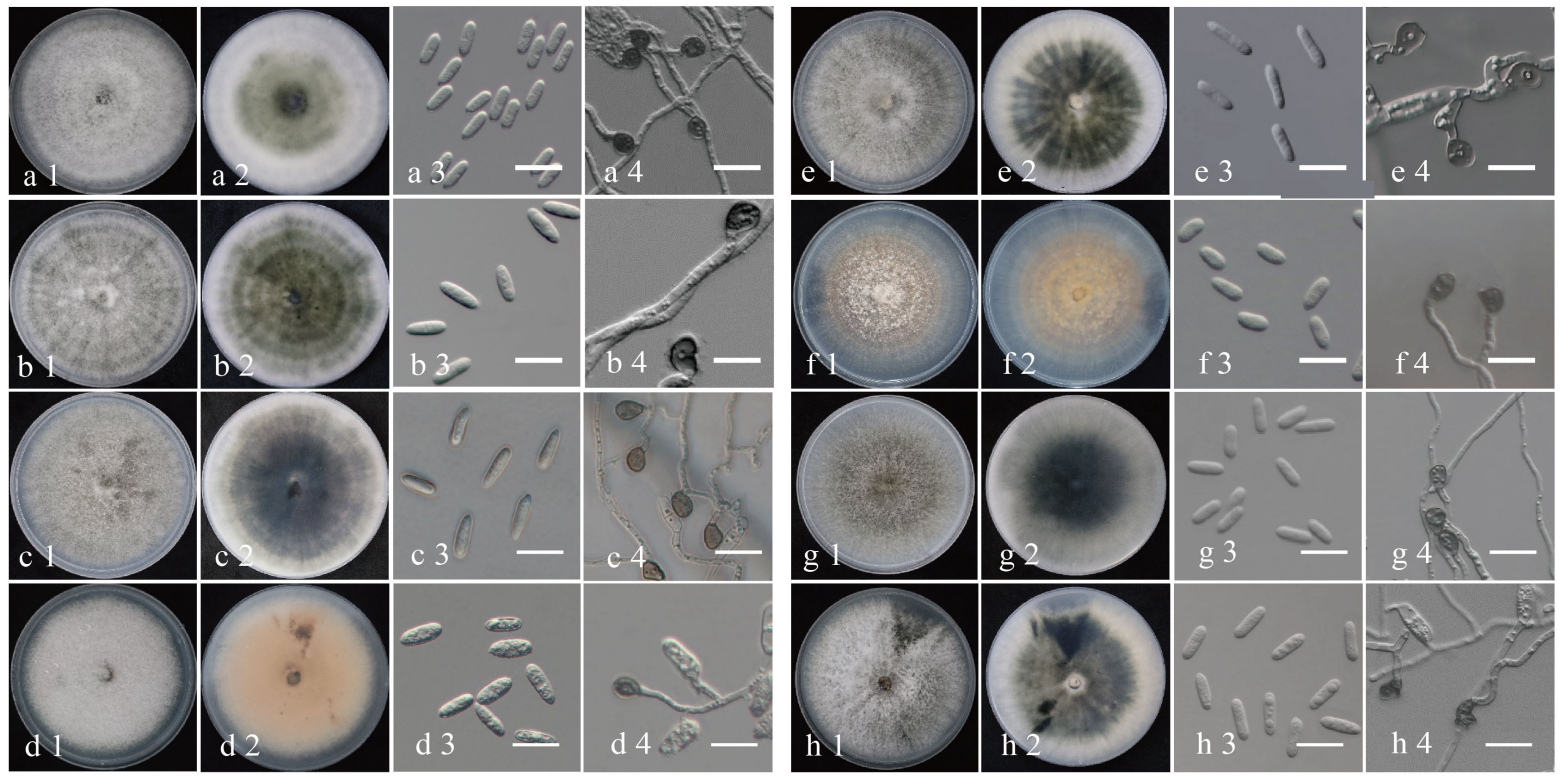
Morphological characteristics of eight *Colletotrichum* isolates from anthracnose leaves of *E*. *japonicus* in Beijing, China. The isolates were cultured on cover slips after 6 dpi at 25°C. **(A)** *C*. *siamense* JZBQ1044–7; **(B)** *C*. *aenigma* JZBQ1044–5; **(C)** *C*. *theobromicola* JZBQ1045–5; **(D)** *C*. *gloeosporioides* JZBQ1373–4; **(E)** *C*. *grossum* JZBQ1264–1; **(F)** *C*. *karstii* JZBQ1115–2; **(G)** *C*. *fructicola* JZBQ978–3; **(H)** *C*. *hebeiense* JZBQ979–2. 1–2: upper and reverse view of the colony; 3: conidia; 4: appressoria. Scale bars = 20 μm.

To further investigate the distinctive characteristics of the eight *Colletotrichum* species, representative isolates were selected from each species for morphological and biological observation (*C. aenigma* JZBQ1044–5, *C. siamense* JZBQ1044–7, *C. theobromicola* JZBQ1045–5, *C. gloeosporioides* JZBQ1373–3, *C. grossum* JZBQ1264–1, *C. karstii* JZBQ1115–2, *C. fructicola* JZBQ978–3 and *C. hebeiense* JZBQ979–2). As shown in **Figure 5**, seven isolates belonging to the *C. gloeosporioides* species complex formed grayish-white colonies on PDA plates with denser and cottony aerial mycelia. On the abaxial surface, all the isolates displayed white margins and gray-black centers, except for *C. gloeosporioides* JZBQ1373–3 which presented a light reddish hue at the center. *Colletotrichum karstii* JZBQ1115–2 exhibited sparse aerial hyphae at the margins and light orange with white flocculent hyphae attached to the center. On the back side, the plate was pale orange in the middle with white margins. The conidia of these isolates exhibited a hyaline, smooth, cylindrical morphology with obtuse rounded ends, or one end was obtusely rounded while the other was more pointed. The conidial widths of *C. karstii* JZBQ1115–2 were comparable to those of the isolates within the *C. gloeosporioides* species complex, while its lengths were significantly shorter (**Table 1**). The appressoria were subglobose or ellipsoid in shape, and possessed intact margins except for *C. hebeiense* JZBQ979–2 which displayed irregular morphology. The mycelial growth rates of *Colletotrichum* spp. exhibited significant variation, with *C. aenigma* JZBQ1044–5 and *C. siamense* JZBQ1044–7 displaying the highest average growth rates, followed by *C. theobromicola* JZBQ1045–5, *C. grossum* JZBQ1264–1 and *C. fructicola* JZBQ978–3. The above results indicate that these eight *Colletotrichum* species exhibited similar morphology of conidia and appressoria, while displaying variations in colony morphology and growth rate.

**Table 1:** Morphological data of eight *Colletotrichum* species on *E. japonicus* in this study.

Species	Isolates	Colony morphology	Growth rate on 25°C (mm/d)	Conidia		Appresoria	
				shape	length × width (μm)	shape	length × width (μm)
*C. siamense*	JZBQ1044–7	Cottony, whitish-grey aerial mycelium; reverse white to black, dark grey in centre	16.3 ± 0.8^a^	Hyaline, smooth-walled, cylindrical, both ends round	14.77 ± 0.74 × 5.23 ± 0.24	Dark brown, regular, but often ellipsoid in outline	9.85 ± 0.74 × 7.70 ± 0.65
*C. aenigma*	JZBQ1044–5	Cottony white aerial mycelium; white to grey; reverse dark grey	16.5 ± 0.5^a^	Hyaline, smooth-walled, cylindrical, both ends round or one end slightly acute	16.23 ± 0.72 × 5.65 ± 0.34	Dark brown, irregular, but often square to ellipsoid in outline	10.22 ± 1.44 × 6.99 ± 0.69
*C. theobromicola*	JZBQ1045–5	Cottony grey aerial mycelium; reverse dark grey	14.0 ± 0.4^b^	Hyaline, smooth-walled, cylindrical, both ends round or one end slightly acute	17.31 ± 1.06 × 6.00 ± 0.66	Dark brown, irregular, but often square to ellipsoid in outline	10.32 ± 0.94 × 7.79 ± 0.89
*C. gloeosporioides*	JZBQ1373–3	Cottony grey-orange aerial mycelium with conidia; reverse dark grey grey-orange and wheel pattern	12.6 ± 0.7^c^	Hyaline, smooth-walled, cylindrical, both ends round or one end slightly acute	17.31 ± 1.24 × 6.45 ± 0.78	Dark brown, regular, often square to ellipsoid in outline	10.34 ± 1.48 × 6.82 ± 0.86
*C. grossum*	JZBQ1264–1	Cottony grey aerial mycelium with angular change; reverse dark grey	14.3± 0.3^b^	Hyaline, smooth-walled, cylindrical, both ends round or one end slightly acute	18.79 ± 2.48 × 5.71 ± 0.63	Dark brown, regular, often ellipsoid in outline	16.82 ± 1.80 × 12.18 ± 1.22
*C. karstii*	JZBQ1115–2	Cottony white-orange aerial mycelium with wheel pattern;white to orange; reverse white to orange	10.6 ± 0.7^c^	Hyaline, smooth-walled, cylindrical, both ends round	13.92 ± 1.15 × 6.37 ± 0.42	Dark brown, regular, often ellipsoid in outline	10.23 ± 1.46 × 8.38 ± 0.87
*C. fructicola*	JZBQ978–3	Cottony grey aerial mycelium; reverse dark grey to black	14.1 ± 0.6^b^	Hyaline, smooth-walled, cylindrical, both ends round	13.50 ± 1.03 × 5.84 ± 0.31	Dark brown, regular, but often ellipsoid in outline	7.67 ± 0.73 × 5.45 ± 0.50
*C. hebeiense*	JZBQ979–2	Cottony white aerial mycelium with conidia and wheel pattern; reverse grey to white with wheel pattern	11.6 ± 0.7^c^	Hyaline, smooth-walled, cylindrical, both ends round	15.17 ± 0.65 × 5.01 ± 0.90	Dark brown, and often irregular in outline	10.62 ± 1.92 × 6.84 ± 0.70

Values are the means ± SD (standard deviation). Letters above the values indicate the significant difference (one-way ANOVA followed by LSD test; p < 0.05). Colony morphology, growth rate, size and morphology of conidia and appressoria were observed and measured on PDA at 25°C for 10 days.

### 3.4 Pathogenicity of *Colletotrichum* spp. on leaves of *E. japonicus*

**Figure 6:**
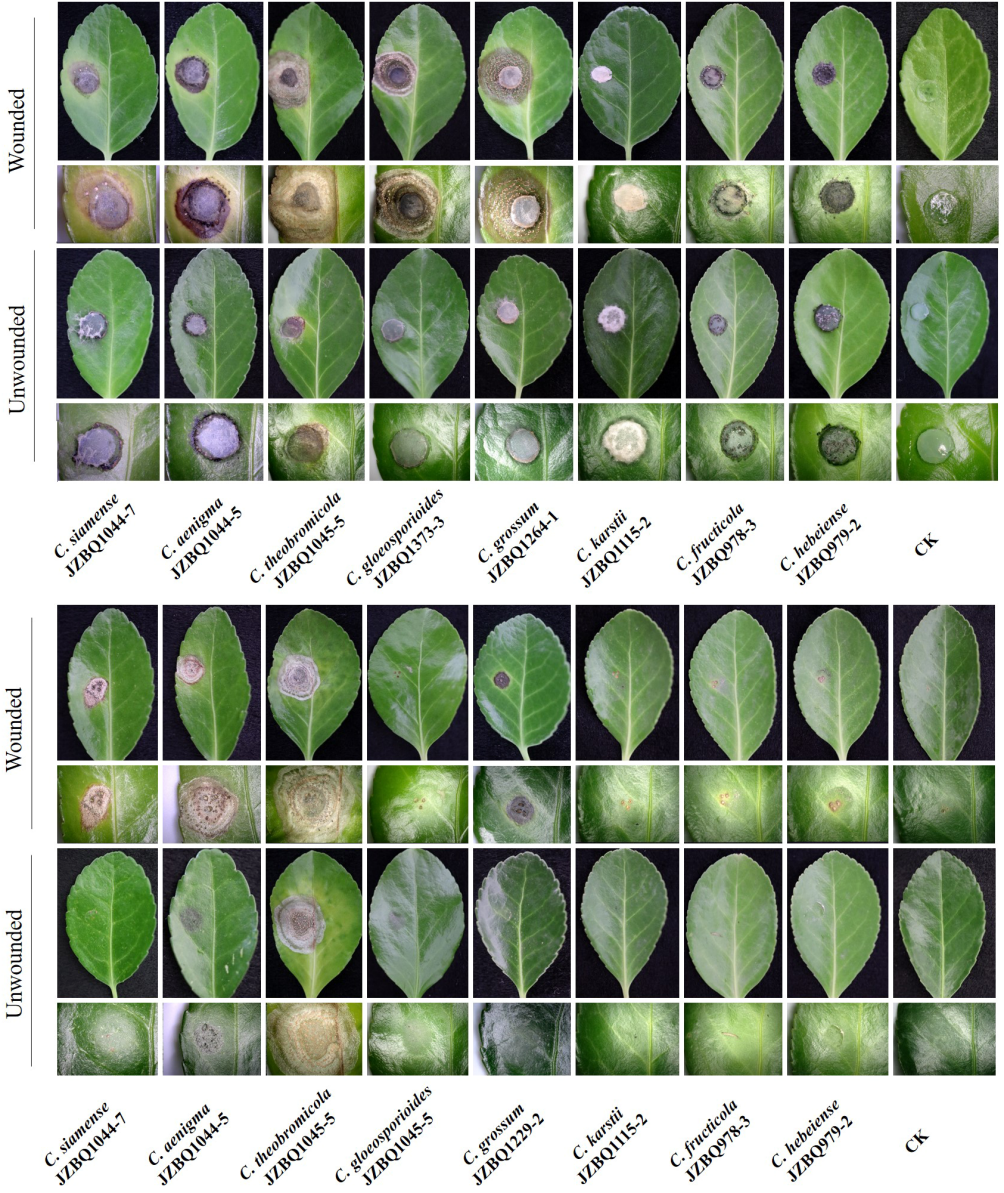
Symptoms on inoculated leaves of *E*. *japonicus* under wounded and unwounded conditions at 7dpi. The inocula were mycelial discs **(A)** or conidial suspensions **(B)** of eight *Colletotrichum* spp.

**Figure 7:**
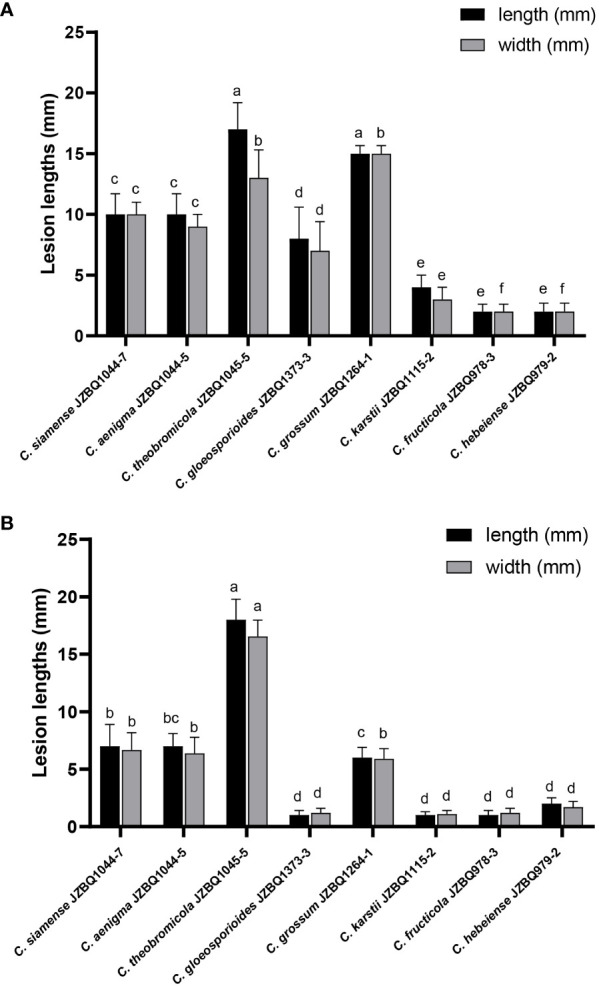
Lesion sizes on wounded leaves of *E*. *japonicus* at 7 dpi inoculated with mycelial discs **(A)** and conidial suspensions **(B)** of eight *Colletotrichum* species. Bars with different lowercase letters indicate significant differences (one-way ANOVA followed by Tukey’s multiple comparison test; p < 0.05).

The pathogenicity of the above eight *Colletotrichum* species was assessed in accordance with Koch’s postulates. After seven days, the leaves inoculated with mycelial discs or conidial suspensions of the eight species under wounded conditions exhibited lesions similar to those observed in the field (**Figure 6**). The control leaves treated with sterile water remained devoid of symptoms. The isolates initiated the development of small brown or dark brown lesions at various time points after inoculation, with symptoms appearing as early as two days post-inoculation (dpi) for *C. aenigma* JZBQ1044–5, *C. siamense* JZBQ1044–7, *C. theobromicola* JZBQ1045–5, *C. gloeosporioides* JZBQ1373–3, and *C. grossum* JZBQ1264–1. These initial lesions gradually expanded into larger brown or dark brown lesions over time and eventually formed concentric rings of acervuli within 3~6 dpi. The pathogenicity of most *Colletotrichum* spp. toward the leaves of *E. japonicus* was found to be dependent on the presence of wounds, as evidenced by the infection rates caused by *Colletotrichum* spp. (**Supplementary Table S5**). Notably, only *C. theobromicola* JZBQ1045–5 and *C. grossum* JZBQ1229–2 were able to cause lesions on leaf tissues in the absence of wounds. Furthermore, the lesion sizes on the wounded leaves of *E. japonicus* at 7 dpi inoculated with mycelial discs of eight *Colletotrichum* spp. showed that *C. theobromicola* JZBQ1045–5 and *C. grossum* JZBQ1264–1 exhibited the highest virulence, while *C. siamense* JZBQ1044–7, *C. aenigma* JZBQ1044–5 and *C. gloeosporioides* JZBQ1373–3 demonstrated moderate levels of virulence; conversely, *C. karstii* JZBQ1115–2, *C. fructicola* JZBQ978–3, and *C. hebeiense* JZBQ979–2 were identified as the least virulent isolates (**Figure 7**). Eight *Colletotrichum* species were identified as potential causative pathogens for anthracnose on *E. japonicus*, with wounds observed to facilitate infection by most of these *Colletotrichum* spp.

### 3.5 Sensitivity of *Colletotrichum* isolates to fungicides

The EC_50_ values were determined by assessing the inhibitory activity of different concentrations of fungicides against *Colletotrichum* isolates. The virulence regression equations and associated data were placed in **Supplementary Table S6**. The results showed that eight fungicides exhibited significant antifungal activity against *C. siamense, C. aenigma*, and *C. theobromicola* (**Table 2**). The EC_50_ values of four triazole fungicides (difenoconazole, flusilazole, tebuconazole, and hexaconazole), as well as prochloraz, against *Colletotrichum* isolates were below 10 mg/L. Among them, prochloraz and difenoconazole were the most effective fungicides. The EC_50_ of prochloraz was less than 0.8 mg/L for the isolates of three species, and even 0.047 mg/L for *C. theobromicola* JZBQ1045–5. The second highly effective fungicide was difenoconazole, with an EC_50_ value as low as 0.2638 mg/mL for *C. theobromicola* JZBQ1388–10. To further observe the effect of fungicides, the colony and mycelial morphology of *C. siamense* JZBQ1044–7, *C. aenigma* JZBQ1044–5 and *C. theobromicola* JZBQ1045–5 treated with fungicides were observed and recorded. All the tested isolates displayed a high degree of sensitivity to various fungicides, and the inhibitory effects on mycelial growth varied significantly among different fungicides. Among them, prochloraz and difenoconazole at high concentrations caused hyphae contraction, increased apical branching, and twisting in *C. siamense* JZBQ1044–7 (**Supplementary Figure S1**). A similar situation was observed after difenoconazole treatment of *C. aenigma* JZBQ1044–5 and *C. theobromicola* JZBQ1045–5. In particular, prochloraz, at a concentration of 0.9 mg/mL, exhibited a significant increase in fungal branching and a decrease in the length of bifurcated hyphae.

**Table 2:** The sensitivity of eight fungicides to the dominant pathogens *C. siamense*, *C. theobromicola*, and *C. aenigma* of *E. japonicas*.

Fungicides	EC_50_ (mg/L)		
	*C. siamense*	*C. aenigma*	*C. theobromicola*
*Difenoconazole*	2.23 ± 0.33^b^	2.49 ± 0.3^b^	0.83 ± 0.59^bc^
*Flusilazole*	2.03 ± 0.77^b^	3.98 ± 1.62^b^	1.37 ± 0.32^bc^
*Tebuconazole*	5.34 ± 1.46^b^	7.99 ± 1.3^b^	0.81 ± 0.34^bc^
*Hexaconazole*	7.71 ± 4.78^b^	8.07 ± 3.47^b^	3.45 ± 1.31^bc^
*Triadimefon*	56.79 ± 17.56^ab^	52.27 ± 4.24^ab^	25.93 ± 4.22^b^
*Prochloraz*	0.41 ± 0.3^b^	0.41 ± 0.08^b^	0.07 ± 0.04^bc^
*Captan*	128.04 ± 67.17^a^	86.88 ± 30.72^a^	66.15 ± 42.75^b^
*Chlorothalonil*	73.91 ± 37.24^a^	66.73 ± 31.35^a^	127.96 ± 59.53^a^

Values are the means ± SD (standard deviation). Letters above the values indicate the significant difference at (one-way ANOVA followed by LSD test; p < 0.05).

## 4 Discussion

*Colletotrichum* spp. as a group are among the top ten fungal pathogens causing wilting and even death of plant parts, as well as post-harvest rotting of fruits worldwide (Dean et al., 2012). For the woody ornamental plant *E. japonicus*, currently, only a few *Colletotrichum* species causing anthracnose, such as *C. siamense*, *C. theobromicola*, and *C. aenigma*, have been reported (Qin et al., 2022; Tan et al., 2023). In this study, pathogenicity tests of eight species *C. aenigma*, *C. siamense*, *C. theobromicola*, *C. gloeosporioides*, *C. grossum*, *C. fructicola*, *C. hebeiense* and *C. karstii* revealed that all eight species significantly infected the leaves of *E. japonicus*. Notably, this study is the first to report that *C. fructicola*, *C. grossum* and *C. hebeiense* cause anthracnose in *E. japonicus* worldwide and that *C. karstii* in China causes anthracnose on *E. japonicus*. Moreover, most isolates belonged to the *C. gloeosporioides* species complex, and *E. japonicus* anthracnose in Beijing was primarily caused by seven species within this complex. This complex comprises 37 species and is associated with numerous plant anthracnose species (Oliveira et al., 2022). For example, various species within this complex, including *C. changpingense*, *C. fructicola*, *C. siamense* and *C. theobromicola*, were found to be responsible for strawberry anthracnose, whereas grape ripe rot in South and North America, Australasia, and Asia was associated with several species such as *C. aenigma*, *C. fructicola*, *C. siamense*, and *C. hebeiense* (Hsieh et al., 2023).

In this study, *C. siamense*, *C. aenigma* and *C. theobromicola* were the three dominant species causing anthracnose on *E. japonicus* in Beijing. *Colletotrichum siamense*, a significant plant pathogen previously documented to induce *E. japonicus* anthracnose in Jiangxi Province, China (Kuang et al., 2022), has been observed to infect numerous other plant species. For example, it can cause anthracnose of Pecan (*Carya illinoinensis)* and *Allamanda cathartica* in China (Huang et al., 2022; Zhuo et al., 2022). Furthermore, it is also the main pathogen causing strawberry crown rot, which has a high prevalence globally and is considered to be one of the most damaging species of the genus *Colletotrichum* (Ji et al., 2022). Similarly, *C. siamense* exhibited strong pathogenicity with a prevalence exceeding 50% in this study. *Colletotrichum theobromicola* has been reported to cause anthracnose of *E. japonicus* (Qin et al., 2022). In this study, pathogenicity test revealed that *C. theobromicola* JZBQ1045–5 exhibited the highest level of pathogenicity, causing significant infection on leaves even in the absence of wounds. This species is also known to induce dieback or anthracnose on a diverse array of plants, such as *Buxus* spp., a commonly used landscape shrub and *Gossypium indicum* (Singh and Doyle, 2017; Kang et al., 2022). The reasons for the high pathogenicity of *C. theobromicola* compared to other species remain to be investigated. *Colletotrichum aenigma* was less pathogenic and less prevalent than *C. siamense* and *C. theobromicola*, but it also exhibits broad host range and significant agricultural affect. For example, *C. aenigma* can cause a leaf spot disease on mulberry, affecting up to 40% of the leaves (Zhu et al., 2022). It can also cause grape anthracnose and apple leaf spot (Kim et al., 2021; Zhang et al., 2021). Therefore, the prevention and control of plant anthracnose caused by *C. siamense*, *C. aenigma*, and *C. theobromicola* are important in both agriculture and urban landscapes.

Most *Colletotrichum* spp. exhibit high pathogenicity toward wounded plants and cause low levels of infection toward unwounded plants. For example, *C. siamense* and *C. fructicola* exhibit increased pathogenicity toward various strawberry cultivars when fruits are damaged (Hu et al., 2022). Additionally, several *Colletotrichum* species are pathogenic to chili peppers following fruit surface injury, but most of them induce only minimal levels of infection in undamaged peppers (De Silva et al., 2019; Hu et al., 2022). The presence of wounds was conducive to the pathogenicity of most *Colletotrichum* spp. tested here to *E. japonicus* either by inoculation with mycelial discs or conidial suspensions. This situation may be explained by inefficient penetration of the pathogen into the cell wall of uninjured plants or by the cuticle acting as an anthracnose infection barrier to impede infection. As a landscape shrub, *E. japonicus* requires frequent pruning. As a result, damage to leaves and branches often occurs, thereby creating favorable conditions for infection by various *Colletotrichum* spp. Therefore, it is crucial to control anthracnose particularly after pruning of *E. japonicus*. As one of strongly pathogenic isolates of *Colletotrichum* spp., *C. theobromicola* exhibits pathogenicity with or without leaf injury supported by reports of *C. siamense* UOM 1137 being strongly pathogenic on both wounded and non-wounded chili peppers (De Silva et al., 2019). Hence, variations in pathogenicity might exist among diverse isolates of *Colletotrichum* spp., necessitating a focus on highly virulent strains.

The application of fungicides remains an important strategy for controlling plant anthracnose. Four triazole fungicides (difenoconazole, flusilazole, tebuconazole, and hexaconazole) had low EC_50_ values against *C. siamense*, *C. aenigma*, and *C. theobromicola*. All of these fungicides, belonging to the demethylation inhibitor (DMI) class, are widely used in agriculture for controlling fungal diseases due to their strong efficacy, and broad spectrum of activity. These fungicides could be used to control phytopathogenic fungi by inhibiting the biosynthesis of ergosterol in the cell membrane of fungi (Liu et al., 2021; Roman et al., 2021). The study revealed that difenoconazole, at a concentration of 0.0319 μg/mL, demonstrates significant inhibitory activity against various *Colletotrichum* species, including *C. siamense*, *C. aenigma*, and *C. fructicola* (Han et al., 2021). Flusilazole has been officially registered for controlling powdery mildew in cucumbers and anthracnose in apples in China (Wang et al., 2020). Tebuconazole exhibits potent inhibitory activity against *Colletotrichum* isolates on tea-oil trees in China, with EC_50_ values ranging from 0.1561 μg/mL to 1.8848 μg/mL (Zhu et al., 2023). Hexaconazole demonstrates effective control against anthracnose disease on pomegranate (*Punica granatum* L.) caused by *C. gloeosporioides* (Raj et al., 2024). These studies suggest the potential application of the four fungicides in managing anthracnose disease in *E. japonicus*. Among the eight fungicides, prochloraz and difenoconazole were the most effective against *C. siamense*, *C. aenigma* and *C. theobromicola*. They exhibit strong control effects against *C. siamense* and *C. fructicola* in the control of strawberry anthracnose (Zhang et al., 2020). Prochloraz is highly inhibitory to a large number of *C. gloeosporioides* species complex isolates (Mora-Aguilera et al., 2021). The imidazole fungicide prochloraz is extensively utilized in horticulture and agriculture worldwide, exerting its inhibitory effect on sterol biosynthesis in fungi (Vinggaard et al., 2006). Therefore, following treatment with prochloraz and difenoconazole, *C. siamense*, *C. aenigma*, and *C. theobromicola* caused twisted and deformed hyphae, as well as shortened bifurcated hyphae, possibly due to restricted cell membrane development. These two fungicides may have potential for the control of anthracnose on *E. japonicus*, but field testing is needed to confirm their effects. In addition, the combination of two multisite fungicides (captan and chlorothalonil) with other fungicides also have great potential applications. For instance, the combined use of captan and paclobutrazol for controlling *C. gloeosporioides* (Pérez, 2020). *In vitro* studies on *C. nymphaeae* and *C. fioriniae* have shown that combinations of two DMI fungicides can exert synergistic effects on *Colletotrichum* species, possibly due to differences in binding with the conserved CYP51 protein (Chen et al., 2020). Furthermore, the alternation and/or combination of single-site and multisite fungicides have been advocated to enhance control efficacy and further prevent the development of resistance in anthracnose isolates (Corkley et al., 2022).

## 5 Conclusions

In this study, a total of 194 *Colletotrichum* isolates were obtained and recognized as eight *Colletotrichum* species responsible for anthracnose of *E. japonicus* during a two-year investigation of 9 districts in Beijing, China. Among them, *C. fructicola*, *C. grossum* and *C. hebeiense* are reported for the first time to be associated with *E. japonicus* anthracnose worldwide, and *C. karstii* is firstly reported to be associated with *E. japonicus* anthracnose in China. Moreover, *C. siamense*, *C. aenigma* and *C. theobromicola* were dominant in the pathogens of anthracnose on *E. japonicus* in Beijing, China. And *C. theobromicola* showed the strongest pathogenicity in the tests. Additionally, the presence of wounds on *E. japonicus* was conducive to the pathogenicity of most *Colletotrichum* spp. whether through inoculation with mycelial discs or conidial suspensions. Therefore, it is crucial to minimize artificial or mechanical wounds to prevent pathogen infection in the production and management of *E. japonicus*. Among eight representative fungicides, four DMI fungicides and prochloraz showed more significant inhibitory effects on *C. siamense*, *C. aenigma*, and *C. theobromicola*, suggesting that they have potential applications toward anthracnose control. These results offer valuable insights for the prevention and treatment of anthracnose of *E. japonicus*.

## Data availability statement

The datasets presented in this study can be found in online repositories. The names of the repository/repositories and accession number(s) can be found in the article/**Supplementary Material**.

## Author contributions

YL: Data curation, Visualization, Writing – original draft, Writing – review & editing. XT: Data curation, Investigation, Methodology, Writing – original draft. JZ: Funding acquisition, Methodology, Writing – review & editing. YN: Data curation, Investigation, Writing – original draft. TH: Methodology, Writing – review & editing. ZY: Conceptualization, Methodology, Writing – review & editing. WQ: Conceptualization, Funding acquisition, Project administration, Writing – review & editing.
