# Supplementary material for: Diversity of Colletotrichum species associated with anthracnose on Euonymus japonicus and their sensitivity to fungicides
Source: Front Plant Sci. 2024 Jun 13;15:1411625. doi: 10.3389/fpls.2024.1411625 (PMC11208684; doi:10.3389/fpls.2024.1411625)
Supplement: Supplementary file 1 [file DataSheet_1.docx]

Supplementary Material

# Supplementary Figures and Tables


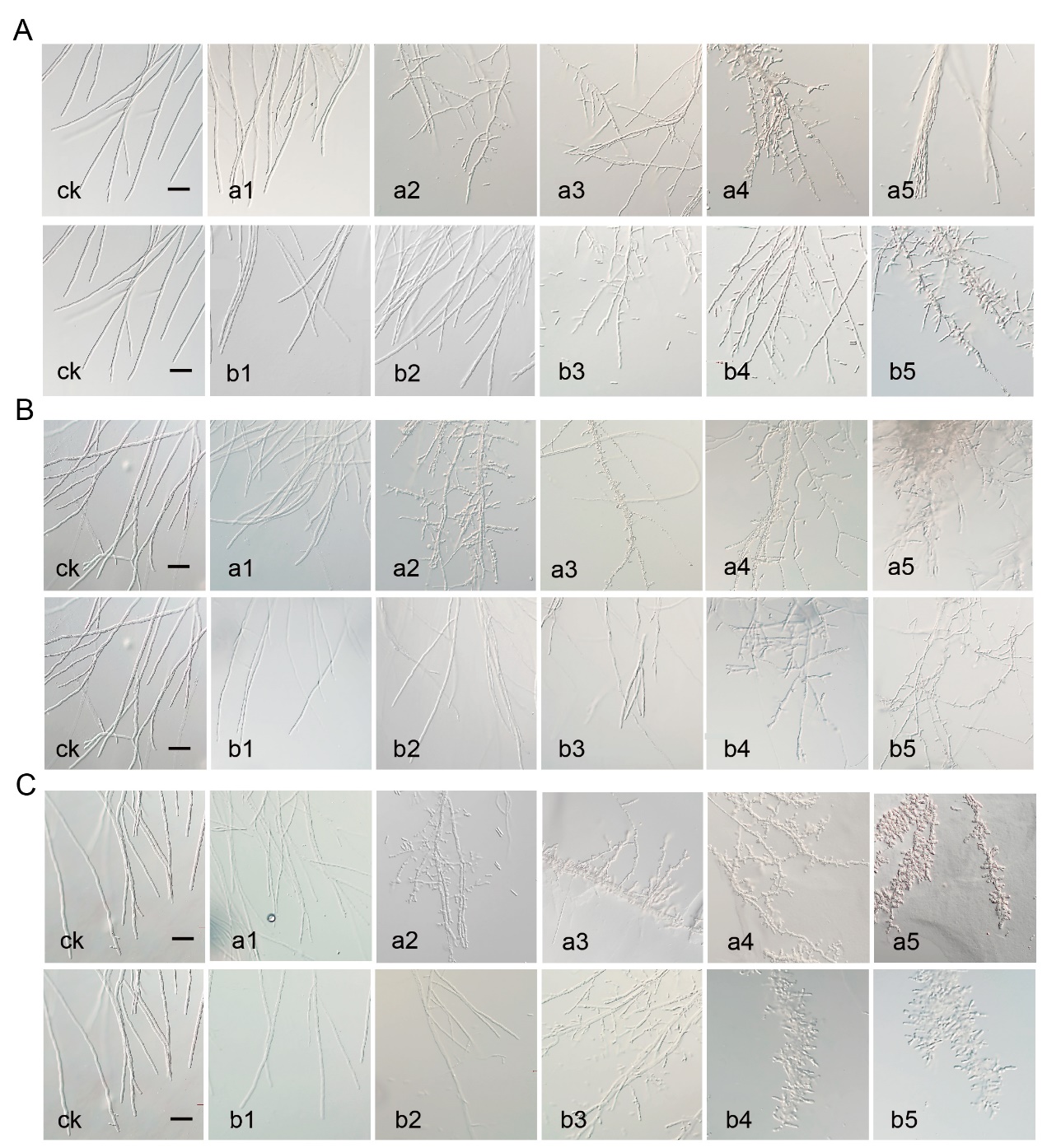


**Supplementary Figure** **S1.** Mycelial microscopic morphologies of *C. siamense* JZBQ1044-7 (A), *C. aenigma* JZBQ1044-5 (B), and *C. theobromicola* JZBQ1045-5 (C) after 6 days of treatment with different concentrations of prochloraz (0.2, 0.9, 4.5, 22.5, and 90 mg/L, respectively, in panels a1-a5) and difenoconazole (0.1, 1, 5, 20, and 50 mg/L, respectively, in panels b1-b5). All scale bars in the images are calibrated according to the ck groups standard, with scale bar=20 μm.

**Table S1** Information including GenBank accession numbers of *Colletotrichum* species used for phylogenetic analysis.

| Species | Strain | GenBank accession numbers | | | | | |
| --- | --- | --- | --- | --- | --- | --- | --- |
|  |  | ITS | *GAPDH* | *CAL* | *ACT* | *CHS-1* | *TUB2* |
| *C. aenigma* | ICMP 18608* | JX010244 | JX010044 | JX009683 | JX009443 | JX009774 | JX010389 |
| *C. aenigma* | ICMP 18686 | JX010243 | JX009913 | JX009684 | JX009519 | JX009789 | JX010390 |
| *C. aeschynomenes* | ICMP 17673* | JX010176 | JX009930 | JX009721 | JX009483 | JX009799 | JX010392 |
| *C. alatae* | CBS 304.67* | JX010190 | JX009990 | JX009738 | JX009471 | JX009837 | JX010383 |
| *C. alienum* | ICMP 12071* | JX010251 | JX010028 | JX009654 | JX009572 | JX009882 | JX010411 |
| *C. artocarpicola* | MFLUCC 18-1167 | MN415991 | MN435568 | –– | MN435570 | MN435569 | MN435567 |
| *C. asianum* | ICMP 18580* | FJ972612 | JX010053 | FJ917506 | JX009584 | JX009867 | JX010406 |
| *C. beeveri* | CBS 128527* | JQ005171 | JQ005258 | JQ005692 | JQ005519 | JQ005345 | JQ005605 |
| *C. boninense* | CBS 123755* | JQ005153 | JQ005240 | JQ005674 | JQ005501 | JQ005327 | JQ005588 |
| *C. brasiliense* | CBS 128501* | JQ005235 | JQ005322 | JQ005756 | JQ005583 | JQ005409 | JQ005669 |
| *C. brassicicola* | CBS 101059* | JQ005172 | JQ005259 | JQ005693 | JQ005520 | JQ005346 | JQ005606 |
| *C. camelliae-japonicae* | CGMCC 3.18118* | KX853165 | KX893584 | –– | KX893576 | –– | KX893580 |
| *C. changpingense* | MFLUCC 15-0022 | KP683152 | KP852469 | –– | KP683093 | KP852449 | KP852490 |
| *C. chrysophillum* | CMM 4268 | KX094252 | KX094183 | –– | KX093982 | KX094083 | KX094285 |
| *C. citricola* | CBS 134228* | KC293576 | KC293736 | KC293696 | KC293616 | KC293696 | KC293656 |
| *C. colombiense* | CBS 129818* | JQ005174 | JQ005261 | JQ005695 | JQ005522 | JQ005348 | JQ005608 |
| *C. conoides* | CAUG17* | KP890168 | KP890162 | KP890150 | KP890144 | KP890156 | KP890174 |
| *C. conoides* | CAUG33 | KP890169 | KP890163 | KP890151 | KP890145 | KP890157 | KP890175 |
| *C. conoides* | CAUG34 | KP890170 | KP890164 | KP890152 | KP890146 | KP890158 | KP890176 |
| *C. constrictum* | CBS 128504* | JQ005238 | JQ005325 | JQ005759 | JQ005586 | JQ005412 | JQ005672 |
| *C. cymbidiicola* | IMI 347923* | JQ005166 | JQ005253 | JQ005687 | JQ005514 | JQ005340 | JQ005600 |
| *C. dacrycarpi* | CBS 130241* | JQ005236 | JQ005323 | JQ005757 | JQ005584 | JQ005410 | JQ005670 |
| *C. dimorphum* | YMF1.07303 | OK030866 | OK513669 | –– | OK513605 | OK513565 | OK513635 |
| *C. endophytica* | MFLUCC 13–0418 | KC633854 | KC832854 | KC810018 | KF306258 | –– | –– |
| *C. fructicola* | ICMP 18613 | JX010167 | JX009998 | JX009675 | JX009491 | JX009772 | JX010388 |
| *C. fructicola* | ICMP 18727 | JX010179 | JX010035 | JX009682 | JX009565 | JX009812 | JX010394 |
| *C. gloeosporioides* | ICMP 17821* | JX010152 | JX010056 | JX009731 | JX009531 | JX009818 | JX010445 |
| *C. gloeosporioides* | ICMP 12939 | JX010149 | JX009931 | JX009728 | JX009462 | JX009747 | –– |
| *C. gloeosporioides* | CBS 112999 | JQ005152 | JQ005239 | JQ005673 | JQ005500 | JQ005326 | JQ005587 |
| *C. grevilleae* | CBS 132879 | KC297078 | KC297010 | KC296963 | KC296941 | KC296987 | KC297102 |
| *C. grossum* | CAUG7* | KP890165 | KP890159 | KP890147 | KP890141 | KP890153 | KP890171 |
| *C. hebeiense* | MFLUCC13–0726* | KF156863 | KF377495 | –– | KF377532 | KF289008 | KF288975 |
| *C. hebeiense* | JZB330289 | OL413586 | OL471174 | OL471176 | OL471178 | OL471180 | OL471182 |
| *C. hebeiense* | JZB330290 | OL413587 | OL471175 | OL471177 | OL471179 | OL471181 | OL471183 |
| *C. hippeastri* | CBS 125376* | JQ005231 | JQ005318 | JQ005752 | JQ005579 | JQ005405 | JQ005665 |
| *C. horii* | ICMP 10492* | GQ329690 | GQ329681 | JX009604 | JX009438 | JX009752 | JX010450 |
| *C. hystricis* | CBS 142411 | KY856450 | KY856274 | KY856103 | KY856023 | KY856190 | KY856532 |
| *C. karstii* | CBS 113087 | JQ005181 | JQ005268 | JQ005702 | JQ005529 | JQ005355 | JQ005615 |
| *C. karstii* | CBS 128551 | JQ005208 | JQ005295 | JQ005729 | JQ005556 | JQ005382 | JQ005642 |
| *C. karstii* | CBS 129824 | JQ005215 | JQ005302 | JQ005736 | JQ005563 | JQ005389 | JQ005649 |
| *C. karstii* | CBS 128552 | JQ005188 | JQ005275 | JQ005709 | JQ005536 | JQ005362 | JQ005622 |
| *C. makassarense* | CBS 143664a | MH728812 | MH728820 | –– | MH781480 | MH805850 | MH846563 |
| *C. nanhuaensis* | YMF1.04993 | OK030870 | OK513673 | –– | OK513609 | OK513569 | OK513639 |
| *C. novae-zelandiae* | CBS 128505* | JQ005228 | JQ005315 | JQ005749 | JQ005576 | JQ005402 | JQ005662 |
| *C. nupharicola* | ICMP 18187* | JX010187 | JX009972 | JX009663 | JX009437 | JX009835 | JX010398 |
| *C. oncidii* | CBS 129828* | JQ005169 | JQ005256 | JQ005690 | JQ005517 | JQ005343 | JQ005603 |
| *C. pandanicola* | MFLUCC 17-0571 | MG646967 | MG646934 | –– | MG646938 | MG646931 | MG646926 |
| *C. parsonsiae* | CBS 128525 | JQ005233 | JQ005320 | JQ005754 | JQ005581 | JQ005407 | JQ005667 |
| *C. parvisporum* | YMF1.06942 | OK030876 | OK513679 | –– | OK513613 | OK513575 | OK513645 |
| *C. perseae* | CBS 141365 | KX620308 | KX620242 | KX620206 | KX620145 | –– | KX620341 |
| *C. petchii* | CBS 378.94* | JQ005223 | JQ005310 | JQ005744 | JQ005571 | JQ005397 | JQ005657 |
| *C. phyllanthi* | CBS 175.67* | JQ005221 | JQ005308 | JQ005742 | JQ005569 | JQ005395 | JQ005655 |
| *C. proteae* | CBS 132882 | KC297079 | KC297009 | KC296960 | KC296940 | KC296986 | KC297101 |
| *C. queenslandicum* | ICMP 1778* | JX010276 | JX009934 | JX009691 | JX009447 | JX009899 | JX010414 |
| *C. salsolae* | ICMP 19051* | JX010242 | JX009916 | JX009696 | JX009562 | JX009863 | JX010403 |
| *C. siamense* | ICMP 18574 | JX010270 | JX010002 | JX009707 | JX009535 | JX009798 | JX010391 |
| *C. siamense* | ICMP 17795 | JX010162 | JX010051 | JX009703 | JX009506 | JX009805 | JX010393 |
| *C. siamense* | ICMP 18642* | JX010278 | JX010019 | JX009709 | GQ856775 | GQ856730 | JX010410 |
| *C. tainanense* | CBS 143666a | MH728818 | MH728823 | –– | MH781475 | MH805845 | MH846558 |
| *C. theobromicola* | ICMP 18649* | JX010294 | JX010006 | JX009591 | JX009444 | JX009869 | JX010447 |
| *C. theobromicola* | ICMP 17895 | JX010284 | JX010057 | JX009600 | JX009568 | JX009828 | JX010382 |
| *C. torulosum* | CBS 128544* | JQ005164 | JQ005251 | JQ005685 | JQ005512 | JQ005338 | JQ005598 |
| *C. tropicale* | CBS 124949* | JX010264 | JX010007 | JX009719 | JX009489 | JX009870 | JX010407 |
| *C. viniferum* | GZAAS 5.08601* | JN412804 | JN412798 | JQ309639 | JN412795 | –– | JN412813 |
| *C. xanthorrhoeae* | ICMP 17903* | JX010261 | JX009927 | JX009653 | JX009478 | JX009823 | JX010448 |
| *C. yunajiangensis* | YMF1.04996 | OK030885 | OK513686 | –– | OK513620 | OK513583 | OK513649 |

Abbreviations: CBS, culture collection of the Centraalbureau voor Schimmelcultures, Fungal Biodiversity Centre, Utrecht, Netherlands; CGMCC, China General Microbiological Culture Collection; ICMP, International Collection of Microorganisms from Plants, Auckland, New Zealand.

* Ex-type culture.

**Table S2 Information of 53 representative isolates of eight *Colletotrichum* spp. isolated from anthracnose leaves of *E. japonicus* in Beijing, China**

| Species | Isolates | Symptoms | Origin | GenBank accession numbers | | | | | |
| --- | --- | --- | --- | --- | --- | --- | --- | --- | --- |
|  |  |  |  | ITS | *GAPDH* | *CAL* | *ACT* | *CHS-1* | *TUB* |
| *C. aenigma* | JZBQ852-1 | Ⅱ | Fangshan | OQ608673 | OQ658066 | OQ676639 | OQ676684 | OQ676729 | OQ676774 |
| *C. aenigma* | JZBQ852-2 | Ⅱ | Fangshan | OQ608674 | OQ658067 | OQ676640 | OQ676685 | OQ676730 | OQ676775 |
| *C. aenigma* | JZBQ852-3 | Ⅱ | Fangshan | OQ608675 | OQ658068 | OQ676641 | OQ676686 | OQ676731 | OQ676776 |
| *C. aenigma* | JZBQ1044-1 | Ⅰ | Haidian | ON637046 | ON667865 | ON667880 | ON667870 | ON667885 | ON667875 |
| *C. aenigma* | JZBQ1044-3 | Ⅰ | Haidian | OQ608676 | OQ658069 | OQ676642 | OQ676687 | OQ676732 | OQ676777 |
| *C. aenigma* | JZBQ1044-5 | Ⅰ | Haidian | ON637047 | ON667866 | ON667881 | ON667871 | ON667886 | ON667876 |
| *C. aenigma* | JZBQ1109-1 | Ⅱ | Chaoyang | OQ608677 | OQ658070 | OQ676643 | OQ676688 | OQ676733 | OQ676778 |
| *C. aenigma* | JZBQ1109-2 | Ⅱ | Chaoyang | OQ608678 | OQ658071 | OQ676644 | OQ676689 | OQ676734 | OQ676779 |
| *C. aenigma* | JZBQ1109-3 | Ⅱ | Chaoyang | OQ608679 | OQ658072 | OQ676645 | OQ676690 | OQ676735 | OQ676780 |
| *C. aenigma* | JZBQ1276-1 | Ⅹ | Dongcheng | OQ608680 | OQ658073 | OQ676646 | OQ676691 | OQ676736 | OQ676781 |
| *C. aenigma* | JZBQ1276-2 | Ⅹ | Dongcheng | OQ608681 | OQ658074 | OQ676647 | OQ676692 | OQ676737 | OQ676782 |
| *C. aenigma* | JZBQ1276-3 | Ⅹ | Dongcheng | OQ608682 | OQ658075 | OQ676648 | OQ676693 | OQ676738 | OQ676783 |
| *C. fructicola* | JZBQ978-3 | Ⅸ | Huairou | OQ608683 | OQ658076 | OQ676649 | OQ676694 | OQ676739 | OQ676784 |
| *C. fructicola* | JZBQ978-5 | Ⅸ | Huairou | OQ608684 | OQ658077 | OQ676650 | OQ676695 | OQ676740 | OQ676785 |
| *C. gloeosporioides* | JZBQ1373-1 | Ⅳ | Haidian | OQ608685 | OQ658078 | OQ676651 | OQ676696 | OQ676741 | OQ676786 |
| *C. gloeosporioides* | JZBQ1373-2 | Ⅳ | Haidian | OQ608686 | OQ658079 | OQ676652 | OQ676697 | OQ676742 | OQ676787 |
| *C. gloeosporioides* | JZBQ1373-3 | Ⅳ | Haidian | OQ608687 | OQ658080 | OQ676653 | OQ676698 | OQ676743 | OQ676788 |
| *C. grossum* | JZBQ1264-1 | Ⅵ | Haidian | OQ608688 | OQ658081 | OQ676681 | OQ676726 | OQ676744 | OQ676816 |
| *C. grossum* | JZBQ1264-2 | Ⅵ | Haidian | OQ608689 | OQ658082 | OQ676682 | OQ676727 | OQ676745 | OQ676817 |
| *C. grossum* | JZBQ1264-3 | Ⅵ | Haidian | OQ608690 | OQ658083 | OQ676683 | OQ676728 | OQ676746 | OQ676818 |
| *C. hebeiense* | JZBQ979-2 | Ⅷ | Huairou | OQ608691 | OQ658084 | OQ676654 | OQ676699 | OQ676747 | OQ676789 |
| *C. hebeiense* | JZBQ979-3 | Ⅷ | Huairou | OQ608692 | OQ658085 | OQ676655 | OQ676700 | OQ676748 | OQ676790 |
| *C. karstii* | JZBQ1115-2 | Ⅴ | Pinggu | OQ613220 | OQ622208 | OQ622212 | OQ622216 | OQ622220 | OQ622224 |
| *C. karstii* | JZBQ1116-1 | Ⅴ | Pinggu | OQ613221 | OQ622209 | OQ622213 | OQ622217 | OQ622221 | OQ622225 |
| *C. karstii* | JZBQ1119-2 | Ⅴ | Pinggu | OQ613222 | OQ622210 | OQ622214 | OQ622218 | OQ622222 | OQ622226 |
| *C. karstii* | JZBQ1120-2 | Ⅴ | Pinggu | OQ613223 | OQ622211 | OQ622215 | OQ622219 | OQ622223 | OQ622227 |
| *C. siamense* | JZBQ544-1 | Ⅰ | Xicheng | OQ608693 | OQ658086 | OQ676656 | OQ676701 | OQ676749 | OQ676791 |
| *C. siamense* | JZBQ544-2 | Ⅰ | Xicheng | OQ608694 | OQ658087 | OQ676657 | OQ676702 | OQ676750 | OQ676792 |
| *C. siamense* | JZBQ544-3 | Ⅰ | Xicheng | OQ608695 | OQ658088 | OQ676658 | OQ676703 | OQ676751 | OQ676793 |
| *C. siamense* | JZBQ984-1 | Ⅰ | Huairou | OQ608696 | OQ658089 | OQ676659 | OQ676704 | OQ676752 | OQ676794 |
| *C. siamense* | JZBQ984-2 | Ⅰ | Huairou | OQ608697 | OQ658090 | OQ676660 | OQ676705 | OQ676753 | OQ676795 |
| *C. siamense* | JZBQ984-3 | Ⅰ | Huairou | OQ608698 | OQ658091 | OQ676661 | OQ676706 | OQ676754 | OQ676796 |
| *C. siamense* | JZBQ1044-7 | Ⅰ | Haidian | ON637049 | ON667868 | ON667883 | ON667873 | ON667888 | ON667878 |
| *C. siamense* | JZBQ1044-8 | Ⅰ | Haidian | ON637050 | ON667869 | ON667884 | ON667874 | ON667889 | ON667879 |
| *C. siamense* | JZBQ1109-7 | Ⅱ | Chaoyang | OQ608699 | OQ658092 | OQ676662 | OQ676707 | OQ676755 | OQ676797 |
| *C. siamense* | JZBQ1109-8 | Ⅱ | Chaoyang | OQ608700 | OQ658093 | OQ676663 | OQ676708 | OQ676756 | OQ676798 |
| *C. siamense* | JZBQ1109-9 | Ⅱ | Chaoyang | OQ608701 | OQ658094 | OQ676664 | OQ676709 | OQ676757 | OQ676799 |
| *C. siamense* | JZBQ1215-1 | Ⅱ | Fengtai | OQ608702 | OQ658095 | OQ676665 | OQ676710 | OQ676758 | OQ676800 |
| *C. siamense* | JZBQ1215-2 | Ⅱ | Fengtai | OQ608703 | OQ658096 | OQ676666 | OQ676711 | OQ676759 | OQ676801 |
| *C. siamense* | JZBQ1215-3 | Ⅱ | Fengtai | OQ608704 | OQ658097 | OQ676667 | OQ676712 | OQ676760 | OQ676802 |
| *C. siamense* | JZBQ1259-1 | Ⅹ | Shijingshan | OQ608705 | OQ658098 | OQ676668 | OQ676713 | OQ676761 | OQ676803 |
| *C. siamense* | JZBQ1259-2 | Ⅹ | Shijingshan | OQ608706 | OQ658099 | OQ676669 | OQ676714 | OQ676762 | OQ676804 |
| *C. siamense* | JZBQ1260-1 | Ⅱ | Shijingshan | OQ608707 | OQ658100 | OQ676670 | OQ676715 | OQ676763 | OQ676805 |
| *C. siamense* | JZBQ1260-4 | Ⅱ | Shijingshan | OQ608708 | OQ658101 | OQ676671 | OQ676716 | OQ676764 | OQ676806 |
| *C. siamense* | JZBQ1274-1 | Ⅱ | Dongcheng | OQ608709 | OQ658102 | OQ676672 | OQ676717 | OQ676765 | OQ676807 |
| *C. siamense* | JZBQ1274-2 | Ⅱ | Dongcheng | OQ608710 | OQ658103 | OQ676673 | OQ676718 | OQ676766 | OQ676808 |
| *C. siamense* | JZBQ1274-3 | Ⅱ | Dongcheng | OQ608711 | OQ658104 | OQ676674 | OQ676719 | OQ676767 | OQ676809 |
| *C. theobromicola* | JZBQ1045-3 | Ⅲ | Haidian | OQ608712 | OQ658105 | OQ676675 | OQ676720 | OQ676768 | OQ676810 |
| *C. theobromicola* | JZBQ1045-4 | Ⅲ | Haidian | OQ608713 | OQ658106 | OQ676676 | OQ676721 | OQ676769 | OQ676811 |
| *C. theobromicola* | JZBQ1045-5 | Ⅲ | Haidian | OQ608714 | OQ658107 | OQ676677 | OQ676722 | OQ676770 | OQ676812 |
| *C. theobromicola* | JZBQ1229-1 | Ⅶ | Haidian | OQ608715 | OQ658108 | OQ676678 | OQ676723 | OQ676771 | OQ676813 |
| *C. theobromicola* | JZBQ1229-2 | Ⅶ | Haidian | OQ608716 | OQ658109 | OQ676679 | OQ676724 | OQ676772 | OQ676814 |
| *C. theobromicola* | JZBQ1229-3 | Ⅶ | Haidian | OQ608717 | OQ658110 | OQ676680 | OQ676725 | OQ676773 | OQ676815 |

**Table S3** Information of fungicides used for sensitivity assays of dominant *Colletotrichum* species.

| Fungicide Type | Name | Manufacturer | Type | Active ingredients content | Concentrations(mg/L) |
| --- | --- | --- | --- | --- | --- |
| Triazoles | Difenoconazole | Syngenta Nantong Crop Protection | WDG | 10% | 0.1，1，5，20，50 |
| Triazoles | Flusilazole | Jiangxi Heyi Chemical | EW | 10% | 1，5，10，20，50 |
| Triazoles | Tebuconazole | Bayer CropScience China | SC | 430 g/L | 2.15，10.75，21.5，107.5，215 |
| Triazoles | Hexaconazole | Shandong Zouping Pesticide | SC | 10% | 2，10，50，100，200 |
| Triazoles | Triadimefon | Shandong Zouping Pesticide | EC | 20% | 2.8，28，140，280，560 |
| Imidazoles | Prochloraz | FMC (Suzhou) Crop Care | EW | 450 g/L | 0.225，0.9，4.5，22.5，50 |
| Organosulfur | Captan | Hebei Guanlong Agrochemical | SC | 40% | 4，40，200，400，800 |
| Aromatics | Chlorothalonil | Qingdao Haina Bio-Tech | SC | 40% | 20，200，500，1000，2000 |

Water dispersible granule (WDG); Emulsion in Water (EW); Suspension concentrate (SC); Emulsifiable Concetrates (EC)

**Table S4** Main symptom types of anthracnose on leaves of *E. japonicus* and the corresponding pathogens

| Type | Typical symptoms | Pathogens |
| --- | --- | --- |
| I | The center of the lesion is white, and the edge is dark brown, following the shape of the conidial mass at both the center and the edge of the lesion. In the early stage, the disease spots appear round or oval, spreading in a punctate pattern, and later connecting and expanding. | *C. aenigma*, *C. siamense* |
| II | The lesion is dark brown in the center and dark brown at the margin, and the center and edge of the lesion follow the conidial mass; The disease spots are round or oval in the early stage, and turned dotted and expanded in the later stage, forming continuous patches. | *C. aenigma*, *C. siamense* |
| Ⅲ | The center of the lesion is white, and the edge is dark brown. The center of the lesion forms a conidial mass, while the edge exhibits an irregular shape. The lesion expands in a punctate manner. | *C. theobromicola* |
| Ⅳ | The lesion is brown, expanding from the center, with a central black conidial mass clustered and distributed. | *C. gloeosporioides* |
| Ⅴ | The disease spots are light brown, and arranged in conidia that expand from point to surface, and the conidia are arranged in concentric chakra. | *C. karstii* |
| Ⅵ | The disease spots are dark brown, with black edges, prone to crusted features, and conidia are born. | *C. grossum* |
| Ⅶ | The lesions appear dark brown, occurring along the leaf margins, with small scattered spots in the center. | *C. theobromicola* |
| Ⅷ | The center of the lesion ranges from light brown to dark red, expanding in a punctate manner. The outer edges of the plant leaves surrounding the lesion fade, and small conidial masses form in the center. | *C. hebeiense* |
| VIIII | The lesions are light brown, with black edges, extending in a punctate manner, and the conidial mass is buried or semi-buried. | *C. fructicola* |
| X | The disease spots are gray, brown on the edges, and spread in a faceted shape, with conidial mass. | *C. aenigma*, *C. grossum*, *C. siamense* |

**Table S5** Infection rates of representative *Colletotrichum* isolates on leaves of *E. japonicus* under different inoculation conditions.

| Inoculum |  | Infection rates (%) | | | |
| --- | --- | --- | --- | --- | --- |
| Species | Isolates | Mycelial discs | | Conidial suspensions | |
|  |  | Wounded | Unwounded | Wounded | Unwounded |
| *C. siamense* | JZBQ1044-7 | 100 | 10 | 100 | 0 |
| *C. aenigma* | JZBQ1044-5 | 100 | 0 | 100 | 0 |
| *C. theobromicola* | JZBQ1045-5 | 100 | 100 | 100 | 100 |
| *C. gloeosporioides* | JZBQ1373-3 | 100 | 0 | 100 | 0 |
| *C. grossum* | JZBQ1264-2 | 100 | 0 | 100 | 20 |
| *C. karstii* | JZBQ1115-2 | 100 | 0 | 100 | 0 |
| *C. fructicola* | JZBQ978-3 | 100 | 0 | 100 | 0 |
| *C. hebeiense* | JZBQ979-2 | 100 | 0 | 100 | 0 |
| Sterile water | - | 0 | 0 | 0 | 0 |

|  | ***C. siamense* JZBQ1044-7** | | | | ***C. siamense* JZBQ1109-7** | | | | ***C. siamense* JZBQ1274-1** | | | |
| --- | --- | --- | --- | --- | --- | --- | --- | --- | --- | --- | --- | --- |
|  | **The virulence regression equation** | **Correlation coefficient R** | **F** | **Std. Error** | **The virulence regression equation** | **Correlation coefficient R** | **F** | **Std. Error** | **The virulence regression equation** | **Correlation coefficient R** | **F** | **Std. Error** |
| **Difenoconazole** | y = 0.5366x + 4.7764 | 0.9744 | 150.80 | 0.04 | y = 0.7184x + 4.7652 | 0.9959 | 365.90 | 0.04 | y = 0.7412x + 4.7817 | 0.9917 | 180.60 | 0.06 |
| **Flusilazole** | y = 0.7446x + 4.9586 | 0.9771 | 169.20 | 0.06 | y = 1.0941x + 4.5599 | 0.9942 | 257.10 | 0.07 | y = 1.0751x + 4.5876 | 0.9898 | 145.80 | 0.09 |
| **Tebuconazole** | y = 0.5677x + 4.6795 | 0.9892 | 365.30 | 0.03 | y = 0.7523x + 4.4142 | 0.9539 | 30.33 | 0.14 | y = 0.6570x + 4.4729 | 0.9878 | 120.80 | 0.06 |
| **Hexaconazole** | y = 0.6175x + 4.7845 | 0.9483 | 71.55 | 0.07 | y = 1.1502x + 3.8546 | 0.9762 | 61.02 | 0.15 | y = 1.2223x + 3.7269 | 0.9957 | 353.50 | 0.07 |
| **Triadimefon** | y = 0.9826x + 3.4239 | 0.9974 | 1576.00 | 0.02 | y = 1.2480x + 2.8274 | 0.9913 | 172.10 | 0.10 | y = 1.2959x + 2.5688 | 0.9997 | 5849.00 | 0.02 |
| **Prochloraz** | y = 0.5113x + 5.0723 | 0.9578 | 89.04 | 0.05 | y = 0.4426x + 5.3895 | 0.9295 | 19.07 | 0.10 | y = 0.5835x + 5.2546 | 0.9388 | 22.30 | 0.12 |
| **Captan** | y = 1.0279x + 2.6252 | 0.9939 | 649.80 | 0.04 | y = 1.1463x + 2.8338 | 0.9751 | 58.18 | 0.15 | y = 1.3728x + 2.2408 | 0.9805 | 74.96 | 0.16 |
| **Chlorothalonil** | y = 0.3797x + 4.4091 | 0.9755 | 157.50 | 0.03 | y = 0.7454x + 3.6007 | 0.9956 | 340.60 | 0.04 | y = 0.7714x + 3.4238 | 0.9927 | 205.90 | 0.05 |

**Table S6** **The virulence regression equation of eight fungicides to the pathogens *C. siamense*, *C. theobromicola*, and *C. aenigma***

|  | ***C. aenigma* JZBQ1044-5** | | | | ***C. aenigma* JZBQ1109-1** | | | | ***C. aenigma* JZBQ1276-1** | | | |
| --- | --- | --- | --- | --- | --- | --- | --- | --- | --- | --- | --- | --- |
|  | **The virulence regression equation** | **Correlation coefficient R** | **F** | **Std. Error** | **The virulence regression equation** | **Correlation coefficient R** | **F** | **Std. Error** | **The virulence regression equation** | **Correlation coefficient R** | **F** | **Std. Error** |
| **Difenoconazole** | y = 0.8843x + 4.6716 | 0.9976 | 626.60 | 0.04 | y = 0.8207x + 4.7046 | 0.9939 | 245.40 | 0.05 | y = 0.8133x + 4.6321 | 0.9933 | 224.90 | 0.05 |
| **Flusilazole** | y = 1.2721x + 4.0246 | 0.9959 | 369.40 | 0.07 | y = 1.2905x + 4.3501 | 0.9963 | 407.70 | 0.06 | y = 0.8725x + 4.5959 | 0.9963 | 403.20 | 0.04 |
| **Tebuconazole** | y = 0.9409x + 4.1938 | 0.9896 | 143.40 | 0.08 | y = 1.1389x + 4.0164 | 0.9840 | 91.59 | 0.12 | y = 1.0447x + 3.9791 | 0.9915 | 176.20 | 0.08 |
| **Hexaconazole** | y = 1.025x + 4.2253 | 0.9980 | 786.20 | 0.04 | y = 1.8646x + 2.9844 | 0.9909 | 162.70 | 0.15 | y = 1.0389x + 4.1573 | 0.9945 | 271.20 | 0.06 |
| **Triadimefon** | y = 1.3251x + 2.7476 | 0.9909 | 163.60 | 0.10 | y = 1.4434x + 2.4637 | 0.9844 | 94.25 | 0.15 | y = 1.3044x + 2.7886 | 0.9839 | 91.24 | 0.14 |
| **Prochloraz** | y = 0.8772x + 5.4195 | 0.9827 | 84.66 | 0.10 | y = 0.7962x + 5.2451 | 0.9801 | 73.44 | 0.09 | y = 0.8513x + 5.3392 | 0.9897 | 144.70 | 0.07 |
| **Captan** | y = 1.1473x + 3.0299 | 0.9380 | 22.00 | 0.24 | y = 1.3548x + 2.2320 | 0.9834 | 88.33 | 0.14 | y = 1.2290x + 2.5520 | 0.9800 | 72.95 | 0.14 |
| **Chlorothalonil** | y = 0.9062x + 3.2366 | 0.9349 | 20.84 | 0.20 | y = 0.8101x + 3.4530 | 0.9493 | 27.40 | 0.15 | y = 0.6006x + 4.1063 | 0.9790 | 69.33 | 0.07 |

|  | ***C. theobromicola* JZBQ1045-5** | | | | ***C. theobromicola* JZBQ221-1** | | | | ***C. theobromicola* JZBQ1388-10** | | | |
| --- | --- | --- | --- | --- | --- | --- | --- | --- | --- | --- | --- | --- |
|  | **The virulence regression equation** | **Correlation coefficient R** | **F** | **Std. Error** | **The virulence regression equation** | **Correlation coefficient R** | **F** | **Std. Error** | **The virulence regression equation** | **Correlation coefficient R** | **F** | **Std. Error** |
| **Difenoconazole** | y = 0.9386x + 5.0955 | 0.9758 | 59.75 | 0.12 | y = 0.7154x + 4.8876 | 0.9857 | 103.10 | 0.07 | y = 0.8163x + 5.4723 | 0.9912 | 168.40 | 0.06 |
| **Flusilazole** | y = 1.3775x + 4.6724 | 0.9989 | 1435.00 | 0.04 | y = 0.5453x + 4.9416 | 0.9454 | 25.26 | 0.11 | y = 1.1937x + 4.9477 | 0.9867 | 111.30 | 0.11 |
| **Tebuconazole** | y = 0.9765x +5.0252 | 0.9665 | 42.65 | 0.15 | y = 0.8951x + 4.9767 | 0.9911 | 166.40 | 0.07 | y = 0.6448x + 5.2379 | 0.9668 | 42.99 | 0.10 |
| **Hexaconazole** | y = 1.3122x + 4.3964 | 0.9723 | 52.10 | 0.18 | y = 1.0881x + 4.5646 | 0.9962 | 397.10 | 0.05 | y = 1.2276x + 4.1471 | 0.9874 | 117.40 | 0.11 |
| **Triadimefon** | y = 1.5115x + 2.9026 | 0.9943 | 262.10 | 0.09 | y = 1.8269x + 2.2832 | 0.9955 | 333.10 | 0.10 | y = 1.4830x + 2.9893 | 0.9978 | 701.80 | 0.06 |
| **Prochloraz** | y = 0.6219x + 5.8253 | 0.9942 | 260.30 | 0.04 | y = 0.5204x + 5.6742 | 0.9733 | 54.11 | 0.07 | y = 0.6593x + 5.6044 | 0.9624 | 37.70 | 0.11 |
| **Captan** | y = 0.7368x + 4.0808 | 0.9604 | 35.74 | 0.12 | y = 1.2815x + 2.5453 | 0.9564 | 32.19 | 0.23 | y = 0.9749x + 3.0565 | 0.9788 | 68.52 | 0.12 |
| **Chlorothalonil** | y = 0.5702x + 3.8637 | 0.9939 | 244.90 | 0.04 | y = 0.9050x + 3.2353 | 0.9962 | 394.20 | 0.05 | y = 0.8590x + 3.0299 | 0.9746 | 56.87 | 0.11 |
